# A positive allosteric modulator of the **β**_1_AR with antagonist activity for catecholaminergic polymorphic ventricular tachycardia

**DOI:** 10.1172/JCI190252

**Published:** 2025-10-16

**Authors:** Alyssa Grogan, Robin M. Perelli, Seungkirl Ahn, Haoran Jiang, Arun Jyothidasan, Damini Sood, Chongzhao You, David I. Israel, Alex Shaginian, Qiuxia Chen, Jian Liu, Jialu Wang, Jan Steyaert, Alem W. Kahsai, Andrew P. Landstrom, Robert J. Lefkowitz, Howard A. Rockman

**Affiliations:** 1 Department of Medicine and; 2 Department of Cell Biology, Duke University Medical Center, Durham, North Carolina, USA.; 3 Division of Cardiology, Department of Pediatrics, Duke University School of Medicine, Durham, North Carolina, USA.; 4 HitGen Inc., Chengdu, China.; 5 VIB-VUB Center for Structural Biology, Brussels, Belgium.; 6 Structural Biology Brussels, Vrije Universiteit Brussels, Brussels, Belgium.; 7 Department of Biochemistry and; 8 Howard Hughes Medical Institute, Duke University Medical Center, Durham, North Carolina, USA.

**Keywords:** Cardiology, Therapeutics, Drug screens, G protein-coupled receptors

## Abstract

Orthosteric beta blockers represent the leading pharmacological intervention for managing heart diseases owing to their ability to competitively antagonize β-adrenergic receptors (βARs). However, their use is often limited by adverse effects such as fatigue, hypotension, and reduced exercise capacity, due in part to nonselective inhibition of multiple βAR subtypes. These challenges are particularly problematic in treating catecholaminergic polymorphic ventricular tachycardia (CPVT), a disease characterized by lethal tachyarrhythmias directly triggered by cardiac β_1_AR activation. To identify small-molecule allosteric modulators of the β_1_AR with enhanced subtype specificity and robust functional antagonism of β_1_AR-mediated signaling, we conducted a DNA-encoded small-molecule library screen and discovered Compound 11 (C11). C11 selectively potentiates the binding affinity of orthosteric agonists to the β_1_AR while potently inhibiting downstream signaling after β_1_AR activation. C11 prevents agonist-induced spontaneous contractile activity, Ca^2+^ release events, and exercise-induced ventricular tachycardia in the CSQ2^–/–^ murine model of CPVT. Our studies demonstrate that C11 belongs to an emerging class of allosteric modulators termed positive allosteric modulator antagonists that positively modulate agonist binding but block downstream function. Its pharmacological properties and selective functional antagonism of β_1_AR-mediated signaling make C11 a promising therapeutic candidate for the treatment of CPVT and other forms of cardiac disease associated with excessive β_1_AR activation.

## Abstract 2

A small molecule β_1_AR allosteric modulator identified by DNA-encoded library screening suppresses arrhythmic activity in a murine model of CPVT.

## Graphical abstract

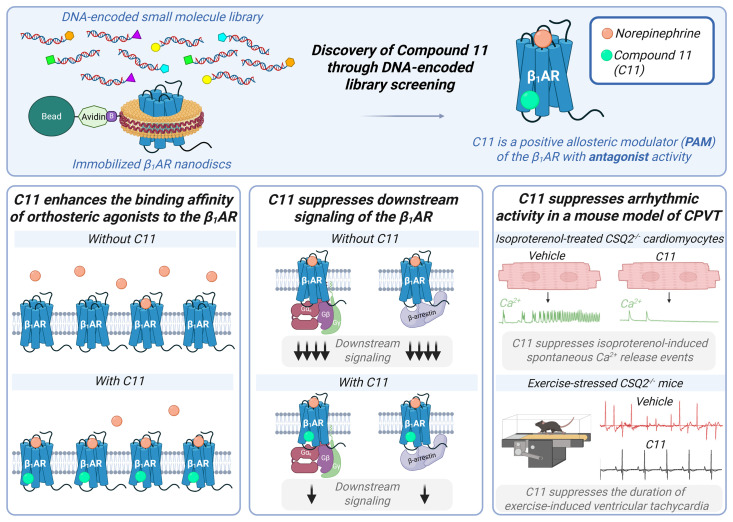

## Introduction

GPCRs are integral regulators of cellular signaling in both health and disease and are readily modulated by an array of molecular modalities, including small molecules, peptides, and hormones (1, 2). Accordingly, GPCRs serve as exemplary therapeutic targets as they are highly amenable to pharmacological modulation and currently comprise more than 30% of all biological entities targeted by FDA-approved drugs (3). As fundamental mediators of the chronotropic and inotropic response in the heart, the β-adrenergic receptor (βAR) subfamily of GPCRs remains one of the most extensively pursued cardiovascular disease targets. βARs are activated via binding of the catecholamine hormone epinephrine and the neurotransmitter norepinephrine to the orthosteric (i.e., endogenous) ligand binding site on the extracellular surface of the receptor. In turn, signaling cascades mediated by heterotrimeric G_s_ and/or β-arrestin transducer proteins are initiated to positively regulate heart rate and contractile dynamics (4). Although acute stimulation of βARs, particularly via the more abundantly expressed cardiac β_1_AR subtype, is an essential physiological response to support increased cardiovascular demand, chronic catecholamine signaling is exceedingly damaging to the heart and is associated with maladaptive morphological remodeling, cardiomyocyte apoptosis, fibrosis, lethal arrhythmias, and heart failure (4).

For decades, traditional orthosteric beta blockers, which competitively antagonize the endogenous ligand binding site of βARs, have been widely used to combat pathological β_1_AR overactivation. Although beta blockers are highly efficacious in reducing morbidity and mortality in heart failure (5), their use is often complicated by adverse effects such as hypotension, fatigue, and reduced exercise capacity that are often associated with the nonselective inhibition of other βAR subtypes, such as the β_2_ARs expressed in vascular and respiratory tissues. These limitations are particularly pertinent to the treatment of catecholaminergic polymorphic ventricular tachycardia (CPVT), a disease characterized by extreme susceptibility to lethal ventricular tachyarrhythmia that develops in direct response to catecholamines (6). To date, nonselective beta blockers such as nadolol and propranolol are the most effective front-line therapy for CPVT (7). However, achieving a maximally tolerated dose without major adverse effects or the occurrence of breakthrough arrhythmias remains a major challenge (5, 8). Thus, CPVT, among other cardiac disorders, could highly benefit from the discovery of efficacious βAR ligands with improved subtype selectivity for the β_1_AR.

Most GPCR-targeting drugs, including beta blockers, bind the orthosteric site, but a rapidly expanding approach to identifying ligands with enhanced specificity, efficacy, and modulatory function is to target allosteric sites of the receptor (9, 10). Allosteric modulators bind to regions that are topographically distinct from the endogenous ligand binding pocket and can increase (positive allosteric modulator, PAM) or suppress (negative allosteric modulator, NAM) the activity of receptors stimulated by an orthosteric ligand. Given that allosteric sites of receptors are more prone to evolutionary divergence and thereby more structurally diverse compared with the orthosteric region that is often highly conserved among closely related receptors of the same family, allosteric modulators are more likely to be subtype-selective with less potential for off-target effects. Moreover, allosteric modulators typically do not possess robust intrinsic activity of their own since they do not directly bind the orthosteric site and should exert a minimal effect on receptor function in the absence of an orthosteric ligand, potentially enabling increased tolerance at higher doses. Also supporting their potential tolerability is the “ceiling effect,” which refers to the saturation of allosteric effects once all binding sites are occupied (11). Together, these properties of allosteric modulators highlight their potential therapeutic advantages relative to their classical orthosteric counterparts (9, 10).

We therefore conducted a DNA-encoded small-molecule library (DEL) screen to identify allosteric modulators of the β_1_AR possessing functional antagonism for use as a therapeutic molecule for treating cardiac diseases. DEL screening is a powerful high-throughput drug discovery technique that enables the simultaneous evaluation of large and chemically diverse libraries where individual molecules are covalently linked to a unique DNA tag. After multiple iterative rounds of affinity selection screening of more than 1 billion unique small molecules comprising the OpenDEL library (HitGen Inc.) against purified functional β_1_ARs reconstituted in lipid nanodiscs, we performed a chemical feature enrichment analysis and identified Compound 11 (C11) as a highly selective allosteric modulator of the β_1_AR. Here, we describe the pharmacological and functional properties of C11 for modulating β_1_AR-mediated signaling and show its therapeutic potential for the treatment of CPVT.

## Results

### Identification of C11 through DNA-encoded small-molecule library screening.

**Figure 1:**
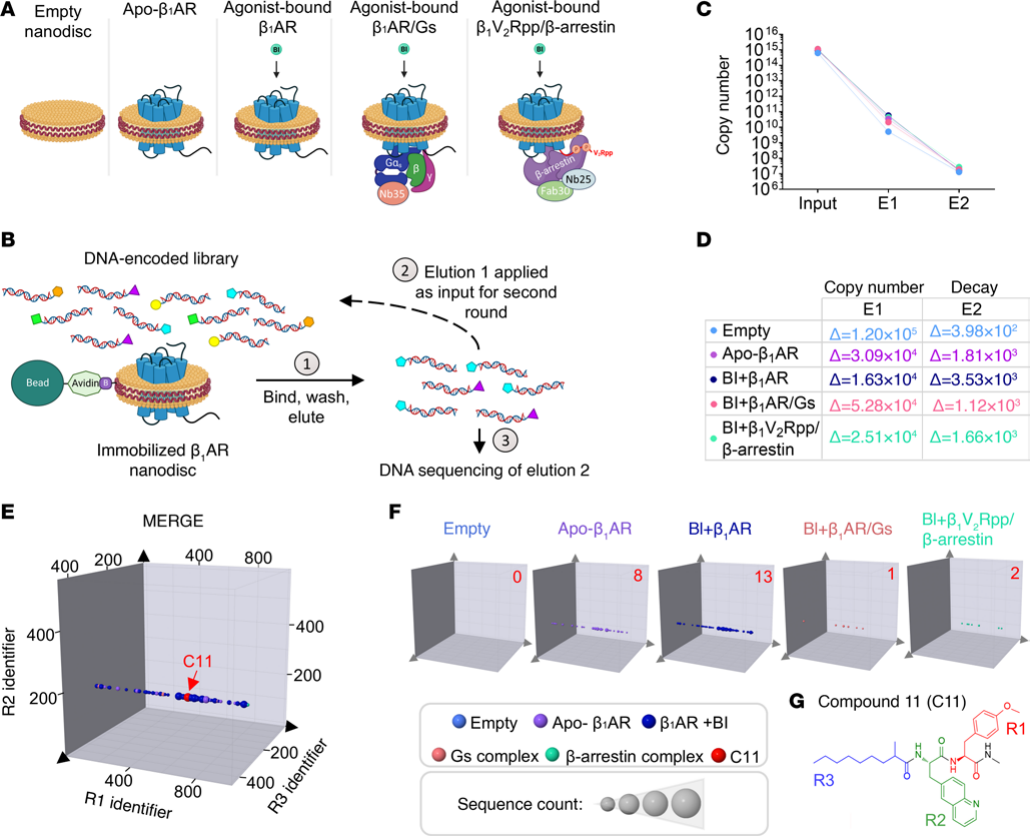
Discovery of C11 through DNA-encoded small-molecule library screening. (**A**) Schematic of screening conditions: empty nanodisc and un-liganded (apo)-β1AR nanodisc controls; β_1_AR nanodiscs bound to high-affinity orthosteric agonist, BI-167107 (BI); and BI-bound β_1_AR or β_1_V_2_Rpp in complex with transducers (heterotrimeric G_s_ or β-arrestin 1, respectively). Transducer complexes were further reinforced using conformation-stabilizing nanobodies Nb35 (included in β_1_AR/G protein complex), Nb25, and Fab30 (included in β_1_V_2_Rpp/β-arrestin complex). (**B**) Purified β_1_ARs reconstituted in biotinylated lipid nanodiscs were immobilized using neutravidin beads and incubated with HitGen’s OpenDEL small-molecule library. After washing, bound compounds were eluted (elution 1) and applied as input for a second round of affinity selection with fresh β_1_AR nanodiscs. Molecules from the final elution (elution 2) were identified by high-throughput DNA sequencing. (**C** and **D**) DNA copy number in eluted samples (E1–E2) was determined by qPCR after each round of affinity selection. Compared with library input (~10^15^ molecules), copy number was reduced to approximately 10^7^ after 2 rounds of screening in each condition (**C**). Approximately 10^4–5^ molecules were lost in round 1 and approximately 10^2–3^ molecules were lost in round 2 (**D**). (**E** and **F**) 3D plots of each screening condition (**F**) and all 5 conditions merged (**E**) depict an enriched chemical line feature — where C11 was identified — which was enriched in Apo-β_1_AR and BI-β_1_AR samples, minimally present in transducer complex samples, and completely absent in the empty nanodisc condition. Axes enumerate the chemical building blocks utilized in each round of chemical synthesis (R1–R3). Data point size corresponds to sequence count for a particular compound; the copy number of C11 in each condition is indicated in red text. Compounds outside of the feature that contains C11 were filtered out to facilitate visualization of the enriched chemotype. (**G**) Schematic of chemical structure of C11 generated through 3 rounds of chemical synthesis: R1, red; R2, green; R3, blue.

To discover allosteric modulators of the β_1_AR with pharmacological and functional properties suitable for potential use as a therapeutic, we conducted a DEL screen using purified β_1_ARs reconstituted in lipid nanodiscs (also known as HDL particles) mimicking the native membrane environment (12). To enable the discovery of molecules that target unique β_1_AR conformational states, we screened 5 conditions: β_1_AR bound to the high-affinity agonist BI-167107 (BI); BI-bound β_1_AR in complex with either heterotrimeric G_s_ or β-arrestin1; and the empty nanodisc and un-liganded β_1_AR (apo-β_1_AR) controls (Figure 1A). Chimeric β_1_ARs harboring the phosphorylated C-terminal tail of the V_2_R (β_1_V_2_Rpp) were engineered to convert β_1_AR to a class B GPCR, thereby strengthening β-arrestin1 complex stability (13) (Supplemental Figure 1, A and B; supplemental material available online with this article; https://doi.org/10.1172/JCI190252DS1). Transducer complexes were further reinforced via conformation stabilizing nanobodies (Nbs) or antibody fragments (Fabs) including Nb35, Nb25, and Fab30 (Figure 1A). Fab30 and Nb25 specifically bind and stabilize the active state of β-arrestin1, and Nb25 enhances the stability of the G protein–bound receptor complex (14–16). Prior to the screen, nanodiscs were functionally validated via radioligand binding (Supplemental Figure 1, C and D), and the affinity selection protocol was optimized as described in the Supplemental Methods and Supplemental Figure 2.

To facilitate the isolation of small-molecule β_1_AR binders from the more than 1 billion unique compounds comprising the OpenDEL library, nanodisc β_1_AR complexes were immobilized to neutravidin beads via biotinylation of the nanodisc membrane scaffold protein, MSPD1E3 (Figure 1B). After 2 consecutive rounds of affinity selection, eluted molecules were purified, PCR amplified, and subjected to high-throughput next-generation sequencing to decode binders (Figure 1B). The decay of library molecules throughout each round of selection was monitored via qPCR using a universal primer set that amplifies all molecules in the library (Figure 1, C and D). In each experimental condition, approximately 1 × 10^7^ molecules were collected in the final elution from the 1 × 10^15^ molecules applied as input (Figure 1, C and D).

Putative hit molecules were identified via comprehensive bioinformatics analysis of the chemical structure similarities among molecules present in the decoded dataset. Enriched chemical features that aligned with our scenario of interest (i.e., an unbiased negative allosteric modulator) were evaluated based on their abundance (or absence) in a particular experimental condition. Using this filtering criteria, we discovered a family of compounds sharing a common partial structure (i.e., R2 and R3) that was significantly enriched in the BI-bound β_1_AR and apo-β_1_AR conditions, minimally present in the G_s_ and β-arrestin1 samples, and completely absent in the empty nanodisc control (Figure 1, E and F). From this family, C11 was selected for further characterization based upon its enrichment profile and relatively high copy number in relation to other members of the compound family (Figure 1, E–G). We predicted that C11 possessed the greatest potential as a β_1_AR negative allosteric modulator, a conclusion that was later corroborated through direct comparison with other structurally related compounds, as detailed below.

### C11 potentiates the binding affinity of agonists and a subset of antagonists to the β_1_AR.

**Figure 2:**
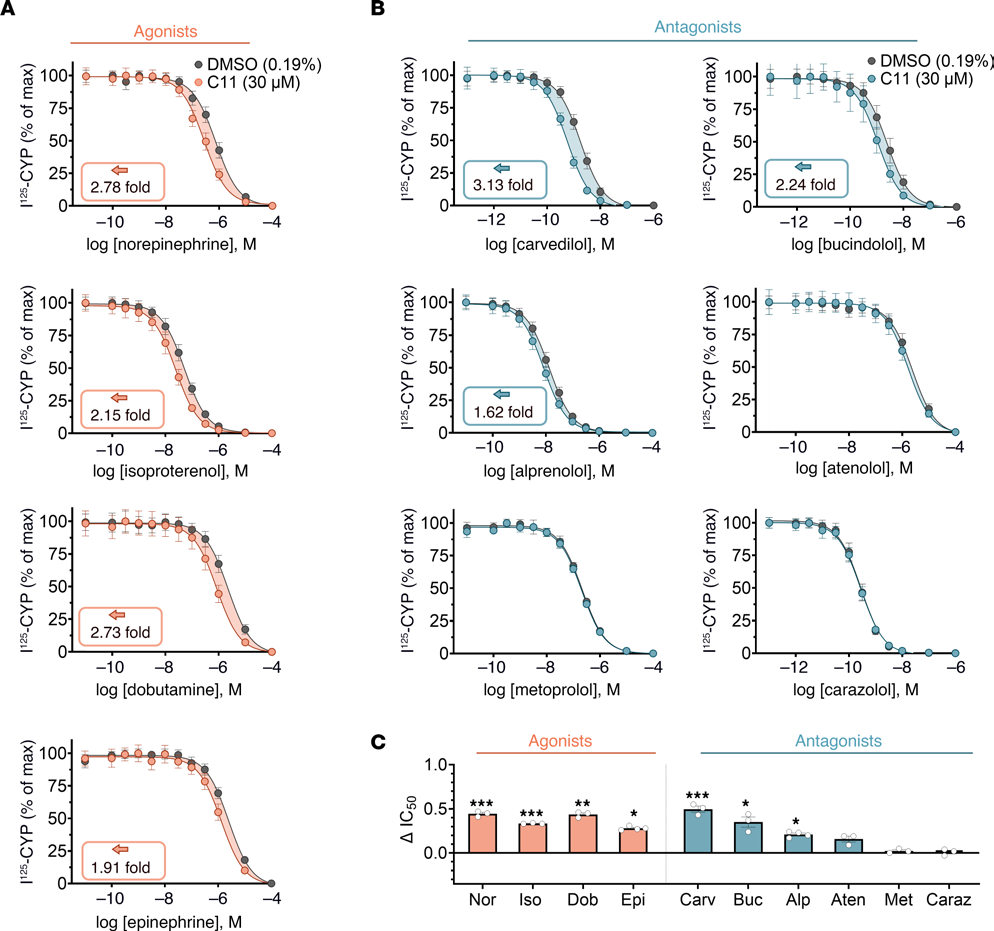
C11 potentiates the binding affinity of agonists and a subset of antagonists to the β_1_AR. β_1_AR nanodiscs were incubated with a fixed amount of radiolabeled orthosteric antagonist, I^125^-CYP; serial doses of unlabeled orthosteric ligand; and either DMSO (0.19%) or 30 μM C11. The resulting competition binding curves (**A** and **B**) and corresponding IC_50_ shift quantifications (**C**) revealed that C11 enhanced the binding affinity of agonists (norepinephrine, Nor; isoproterenol, Iso; dobutamine, Dob; and epinephrine, Epi) and a subset of antagonists (carvedilol, Carv; bucindolol, Buc; alprenolol, Alp) to the β_1_AR with no effect on the binding affinity of the antagonists atenolol (Aten), metoprolol (Met), or carazolol (Caraz). Dose-response curves are presented as percentage of maximum I^125^-CYP binding. IC_50_ values were calculated from the nonlinear fit (1-site binding; GraphPad Prism) and plotted as the difference between IC_50_ (DMSO) and IC_50_ (C11). Raw IC_50_ values are presented in Supplemental Table 1. *F* tests were performed on the nonlinear fit; *P < 0.05, **P < 0.01, ***P < 0.001; data points represent mean ± SEM of at least 3 independent experiments performed in duplicate.

To interrogate the pharmacological properties of C11 on the β_1_AR, we investigated the ability of C11 to modulate orthosteric ligand binding affinity via radioligand competition binding experiments. Purified β_1_AR nanodiscs were incubated with the radiolabeled β_1_AR antagonist, ^125^I-CYP, and serial concentrations of un-labeled orthosteric β_1_AR agonists or antagonists. In the presence of C11, the binding affinity of norepinephrine, isoproterenol, dobutamine, and epinephrine for the β_1_AR was significantly enhanced, as evidenced by an approximately 0.4-log leftward shift and corresponding approximately 1.9-fold to 3-fold decrease in the IC_50_ of the competition binding curve compared with the vehicle control (Figure 2, A and C, and Supplemental Table 1), demonstrating positive cooperativity between C11 and β_1_AR agonists. Interestingly, C11 exhibited a probe-dependent effect with respect to orthosteric antagonist binding to the β_1_AR since it potentiated the binding of a subset of antagonists or biased ligands (i.e., carvedilol, bucindolol, and alprenolol; up to 0.5-log leftward shift and ~3-fold IC_50_ decrease) without affecting atenolol, metoprolol, or carazolol binding affinity (Figure 2, B and C, and Supplemental Table 1). This indicates that the effect of C11 on orthosteric ligand binding to the β_1_AR is specific to the ligand bound. Overall, these binding results are consistent with our screening output in which C11 was enriched in not only the inactive apo-β_1_AR state but also in BI-occupied active β_1_AR condition.

To estimate the affinity of C11 for the β_1_AR, the C11-mediated concentration-dependent increase in isoproterenol binding to the receptor (quantified as ΔIC_50_ of the competition binding curve) was plotted as a function of increasing concentrations of C11 (Supplemental Figure 3, A–C). The resulting logEC_50_ (–6.12, ~0.76 μM) derived from the nonlinear fit suggests sub-micromolar binding affinity between C11 and the β_1_AR in the presence of isoproterenol (Supplemental Figure 3, A–C). In agreement with this finding, direct assessment of the physical interaction and binding affinity between C11 and active β_1_AR bound to high-affinity agonist BI by isothermal titration calorimetry (ITC) revealed a *K_D_* of 4.75 μM (Supplemental Figure 3D).

### C11 suppresses G protein and β-arrestin signaling downstream of agonist-activated β_1_AR.

**Figure 3:**
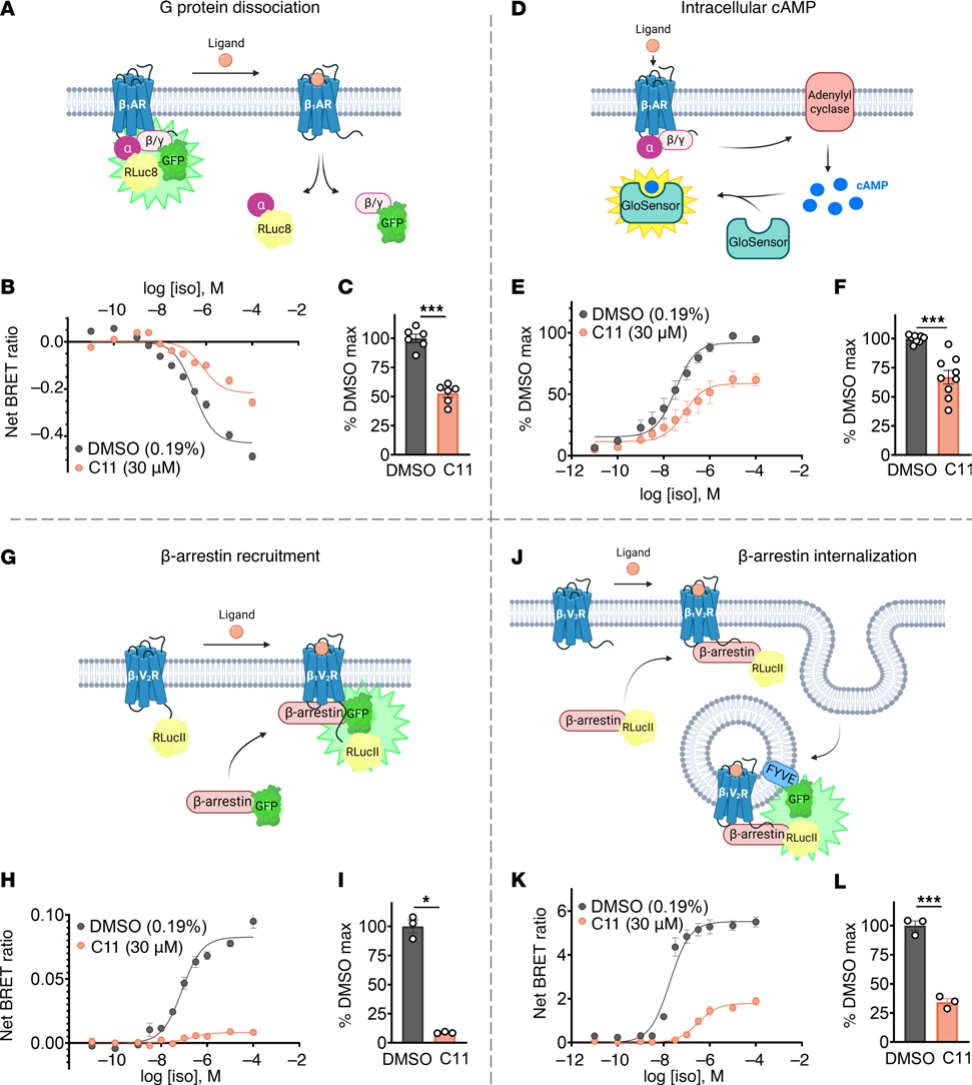
C11 suppresses G protein signaling and β-arrestin function downstream of agonist-activated β_1_AR in HEK293T cells. (**A**–**L**) HEK293T cells transiently transfected with β_1_AR (or β_1_V_2_R) and BRET or luciferase biosensor plasmids were pretreated with DMSO (0.19%) or 30 μM C11 and then stimulated with serial doses of the agonist isoproterenol (Iso). Schematic representation of the BRET-based TRUPATH G protein dissociation assay where Gα_s_-RLuc8 dissociates from Gβ/Gγ-GFP upon β_1_AR activation, resulting in BRET signal decay (**A**). C11 treatment significantly reduced maximal G protein dissociation compared with vehicle (**B** and **C**). Schematic representation of the GloSensor luciferase-based biosensor that emits light in response to binding intracellular cAMP (**D**). C11 treatment significantly reduced maximal cAMP accumulation compared with vehicle (**E** and **F**). Schematic representation of the BRET-based β-arrestin recruitment assay wherein β-arrestin–GFP is recruited to β_1_V_2_R-RLucII upon receptor activation and generates a BRET signal (**G**). C11 treatment significantly reduced maximal β-arrestin recruitment to β_1_V_2_R-RLucII compared with vehicle (**H** and **I**). Schematic representation of BRET-based β-arrestin–mediated receptor internalization assay (**J**). Upon β_1_V_2_R activation, the receptor/β-arrestin–RLucII complex is internalized into endosomes and a BRET signal is generated when internalized β-arrestin–RLucII and endosomal marker FYVE-GFP are in proximity. C11 treatment significantly reduced maximal β-arrestin internalization compared with vehicle (**K** and **L**); *F* test, *P < 0.05, ***P < 0.001; data points represent mean ± SEM of at least 3 independent experiments performed in duplicate or quadruplicate; curve fits were plotted using a log(agonist) 3-parameter model in GraphPad Prism; net BRET ratios (emission of RLuc8/GFP) are baseline-subtracted according to the nonlinear fit of each treatment condition; luminescence values and E_max_ quantifications (derived from the nonlinear fit) are presented as the percentage of maximal signal in the vehicle condition.

Given the positive cooperativity between C11 and orthosteric agonist binding to the β_1_AR, we anticipated that C11 would enhance β_1_AR downstream signaling functioning as a PAM. The functional impact of C11 on G protein and β-arrestin signaling in response to β_1_AR activation was assessed via bioluminescence resonance energy transfer–based (BRET-based) and/or luciferase-based cellular signaling assays. To measure dissociation of Gα_s_βγ upon β_1_AR stimulation, HEK293T cells transiently overexpressing β_1_AR with TRUPATH biosensor proteins Gα_s_-RLuc8, Gβ3, and Gγ9-GFP (17) were pretreated with vehicle (DMSO) or 30 μM C11 and stimulated with serial concentrations of isoproterenol (Figure 3A). Surprisingly, despite its positive cooperativity with agonist binding, C11 substantially reduced maximal G protein dissociation (Figure 3, B and C), as evidenced by an attenuation of the BRET signal decay. To measure Gα_s_-mediated signaling downstream of agonist-activated β_1_ARs, we quantified intracellular cAMP generation utilizing the luciferase-based GloSensor cAMP biosensor (Figure 3D). Consistent with our TRUPATH results, pretreatment with C11 significantly diminished maximal cAMP generation compared with vehicle-treated cells (Figure 3, E and F).

Utilizing similar BRET-based approaches, we evaluated the effect of C11 on the various functions of β-arrestin. To measure β-arrestin recruitment to agonist-activated β_1_AR, we developed a BRET sensor pair consisting of β-arrestin2-GFP and a chimeric β_1_AR containing the C-terminal tail of the V_2_R (β_1_V_2_R) conjugated to RLucII (Figure 3G and Supplemental Table 2) that was utilized to enhance β-arrestin affinity to agonist-occupied β_1_ARs. To measure β-arrestin–mediated receptor internalization, β_1_V_2_R was coexpressed with β-arrestin2-RLucII and the early endosomal marker FYVE-rGFP (18) (Figure 3J). Strikingly, pretreatment with C11 induced a robust decrease in both β-arrestin recruitment to β_1_V_2_R (Figure 3, H and I) and β-arrestin–mediated receptor internalization into endosomes (Figure 3, K and L). These findings indicate that C11 acts as an unbiased functional NAM of agonist-activated β_1_AR since it potently inhibits both G_s_ and β-arrestin signaling.

**Figure 4:**
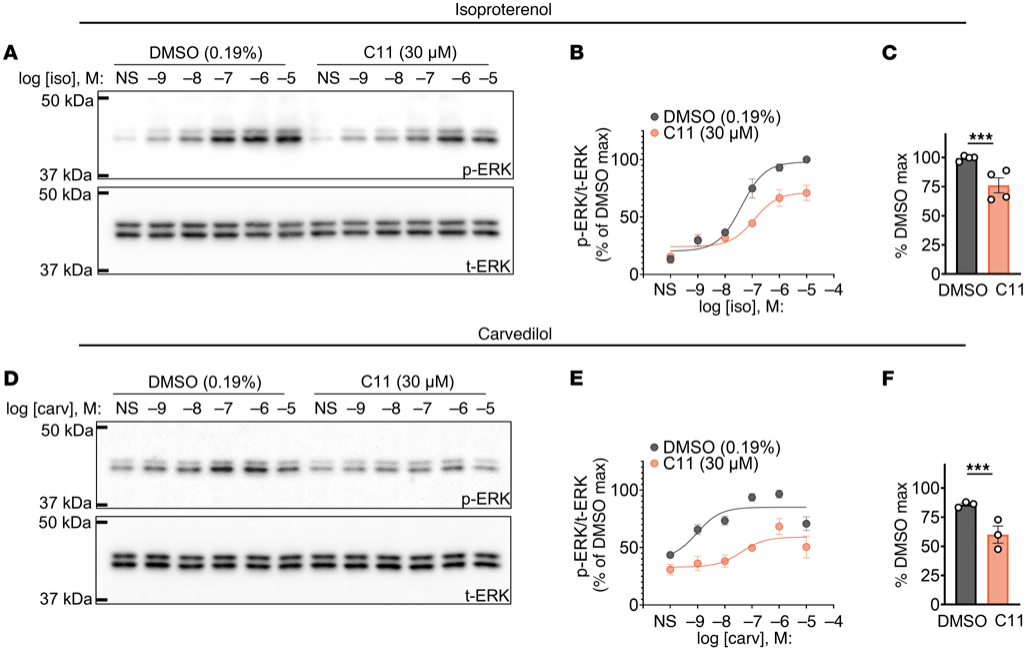
C11 suppresses phosphorylation of ERK mediated by isoproterenol or carvedilol downstream of agonist-activated β_1_AR in HEK293T cells. (**A**–**C**) Representative immunoblots (**A**), relative densitometry quantifications (**B**), and E_max_ values of the nonlinear curve fit (**C**) demonstrated that C11 significantly reduced maximal ERK phosphorylation (pERK) in response to isoproterenol (Iso) compared with vehicle control in HEK293T cells transiently expressing the β_1_AR. (**D**–**F**) Representative immunoblots (**D**), relative densitometry quantifications (**E**), and E_max_ values of the nonlinear curve fit (**F**) demonstrated that C11 significantly reduced maximal pERK in response to carvedilol (Carv) compared with vehicle control in HEK293T cells transiently expressing β_1_AR; densitometric values of pERK were normalized to total ERK (tERK) and presented as the percentage of the maximal value in vehicle-treated cells; *F* test, ***P < 0.001; data points represent mean ± SEM of at least 3 independent experiments; nonlinear curve fits and E_max_ values were calculated from a log(agonist) 3-parameter model in GraphPad Prism.

Given that ERK is one of the major cellular effectors of both G_s_ and β-arrestin signaling cascades, we next evaluated the effect of C11 on β_1_AR-mediated ERK phosphorylation via immunoblotting. Consistent with our BRET-based assays, HEK293T cells transiently overexpressing β_1_AR displayed a dose-dependent increase in ERK phosphorylation in response to serial concentrations of isoproterenol that was suppressed in cells pretreated with C11 (Figure 4, A–C). Notably, ERK phosphorylation stimulated by carvedilol, a β-arrestin–biased βAR ligand that has been previously shown to promote ERK phosphorylation in a β-arrestin–dependent manner (19–22), was also attenuated in the presence of C11 (Figure 4, D–F). This indicates that C11 serves as a functional NAM of the β_1_AR irrespective of the nature of the orthosteric ligand (i.e., full agonists versus biased ligands).

Taken together, in striking opposition to the positive cooperativity of agonist binding, our cellular signaling assays revealed that C11 is a potent inhibitor of β_1_AR-mediated Gα_s_ and β-arrestin signaling. Moreover, its suppression of maximal β_1_AR signaling efficacy reflects the classical pattern of noncompetitive inhibition, as would be expected from an allosteric modulator. With such a unique pharmacological and functional profile, our studies indicate that C11 likely belongs to a recently established class of allosteric modulators termed PAM antagonists that potentiate agonist affinity for receptors while antagonizing downstream signaling (23). Although largely under-characterized to date, PAM antagonists are predicted to be especially favorable therapeutically due to their positive cooperativity on agonist binding affinity (23).

### The PAM-antagonist function of C11 is highly selective for β_1_ARs.

**Figure 5:**
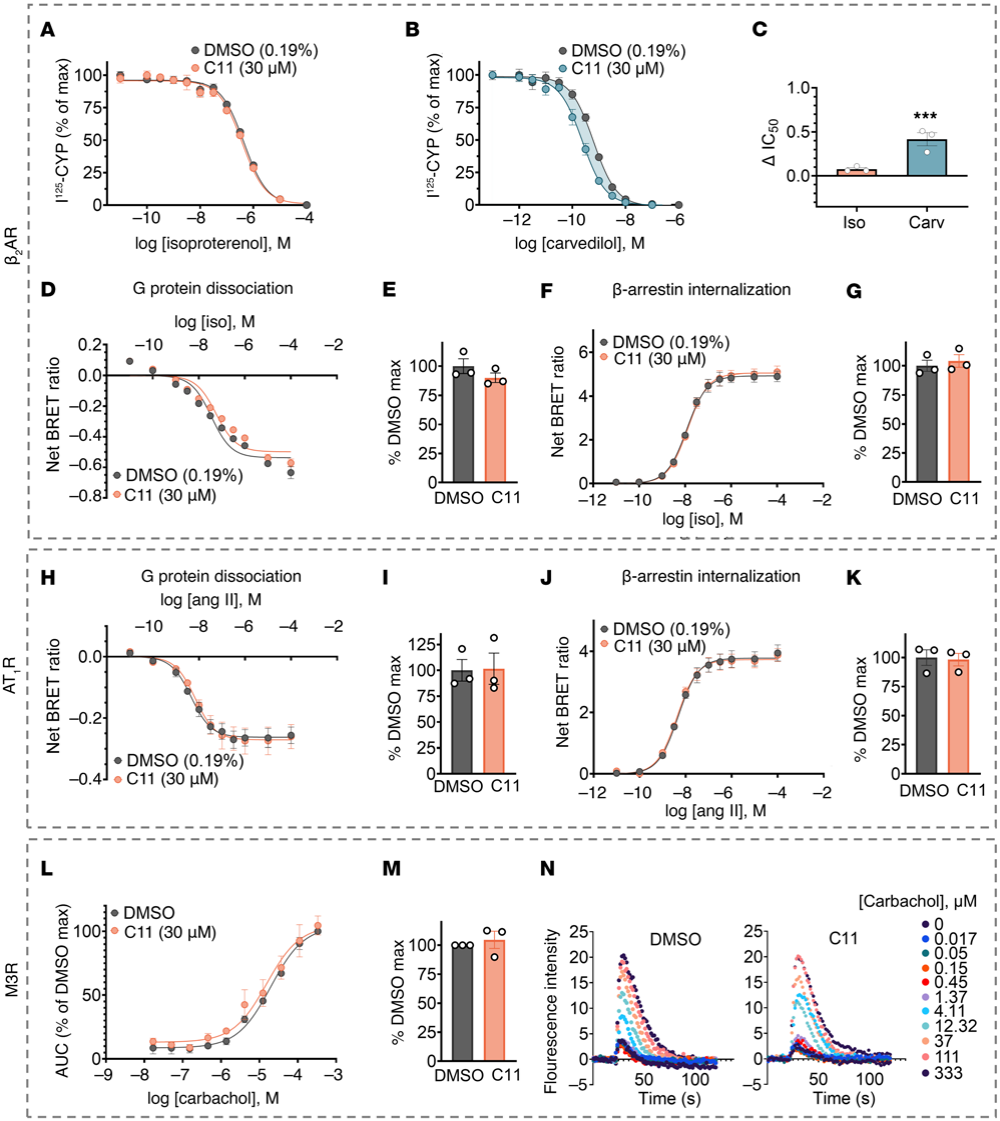
C11 does not suppress cellular signaling mediated by alternative receptors β_2_AR, AT1R, or M3R. (**A**–**C**) β_2_AR nanodiscs were incubated with a fixed amount of radiolabeled orthosteric antagonist, I^125^-CYP; serial doses of unlabeled orthosteric ligand; and either vehicle or 30 μM C11. Competition binding curves (**A** and **B**) and corresponding IC_50_ shifts (**C**) revealed that C11 enhanced the binding affinity of carvedilol (Carv) to the β_2_AR but not isoproterenol (Iso). Dose-response curves are presented as percentage of maximum I^125^-CYP binding. IC_50_ values were calculated from the nonlinear fit (1-site binding; GraphPad Prism) and plotted as the difference between IC_50_ (DMSO) and IC_50_ (C11). *F* tests were performed on the nonlinear fit; ***P < 0.001. Data points represent mean ± SEM of at least 3 independent experiments performed in duplicate. (**D**–**K**) HEK293T cells transiently overexpressing receptor (β_2_AR, β_2_V_2_R, or AT1R) and BRET fusion proteins (Figure 3, A and J) were pretreated with vehicle or 30 μM C11 and stimulated with either isoproterenol (for β_2_AR or β_2_V_2_R) or angiotensin II (Ang II, for AT1R). C11 treatment had no significant effect on G-protein dissociation or β-arrestin internalization downstream of agonist-activated β_2_AR/β_2_V_2_R (**D**–**G**) or AT1R (**H**–**K**). (**L**–**N**) HEK293T cells pretreated with vehicle or 30 μM C11 were stimulated with carbachol to activate the endogenous G_q_-coupled muscarinic M3 receptor (M3R). C11 treatment had no significant effect on the resulting Ca^2+^ response (**L**–**M**). Representative time-course plots of the baseline-subtracted raw fluorescence at each carbachol dose depicted comparable Ca^2+^ responses between vehicle- and C11- treated cells (**N**); data points represent mean ± SEM of at least 3 independent experiments performed in duplicate or triplicate; curve fits were plotted using a log(agonist) 3-parameter model in GraphPad Prism; net BRET ratios (emission of RLuc8/GFP) are baseline-subtracted according to the nonlinear fit of each treatment condition. Ca^2+^ responses are presented as the baseline-subtracted AUC and normalized to the percentage of DMSO maximum.

We next evaluated the pharmacological selectivity of C11 for the β_1_AR by interrogating its effect on orthosteric ligand binding to the β_2_AR subtype. Radioligand binding experiments utilizing β_2_AR nanodiscs demonstrated that C11 did not significantly alter the isoproterenol binding curve compared with vehicle, whereas carvedilol binding to the β_2_AR was potentiated by approximately 2.6-fold (~0.4-log leftward shift) in the presence of C11 (Figure 5, A–C). These data indicate that C11 also binds and modulates ligand binding to the β_2_AR, given that it potentiates carvedilol binding to both subtypes, but does not elicit an increase in affinity between β_2_AR and the agonist isoproterenol. Of note, this βAR subtype–dependent activity is reminiscent of the recently discovered β_2_AR positive allosteric modulator, Compound 6, that potentiates carvedilol binding to both β_1_AR and β_2_AR while selectively increasing agonist binding to the β_2_AR only (22, 24, 25).

To determine the functional selectivity of C11 for the β_1_AR, we performed various counter-assays interrogating G protein and β-arrestin signaling stimulated by alternative GPCRs of the cardiovascular system including the β_2_AR, AT1R, and M3R. Importantly, C11 did not significantly affect G protein dissociation or β-arrestin internalization downstream of either agonist-activated β_2_AR (Figure 5, D–G) or AT1R (Figure 5, H–K). Moreover, C11 had no effect on intracellular Ca^2+^ release downstream of carbachol-activated endogenous M3R (Figure 5, L–N). Together, these experiments indicate that the functional effects of C11 are highly selective for the β_1_AR subtype.

### C11 is a superior PAM antagonist relative to structurally related OpenDEL analogs.

To gain mechanistic insights into the chemical features that support the pharmacological and functional effects of C11 on the β_1_AR and to evaluate whether small chemical modifications in its structure would confer altered efficacy as a PAM antagonist, we characterized a panel of C11 analogs C11-A through C11-I (Supplemental Figure 4) and evaluated their effects on ligand binding and β_1_AR-mediated signaling. These analogs comprise additional members of the enriched family of structurally related OpenDEL molecules detected via our bioinformatics analysis (Figure 1, E and F; C11-A through C11-F and C11-H) as well as several truncated derivatives (C11-G and C11-I). The ability of C11 to potentiate agonist and antagonist binding to the β_1_AR was largely unaffected by modifying the R1 chemical group, given the lack of substantial differences in the IC_50_ shifts between C11 and analogs A–F and H (Supplemental Figure 5, A–C). Remarkably, truncation of the extended hydrocarbon chain comprising the R3 chemical group in C11 and C11-H (generating C11-G and C11-I, respectively) resulted in complete loss of the positive cooperativity of isoproterenol and carvedilol binding to the β_1_AR (Supplemental Figure 5, A–C). These results were corroborated by evaluation of Gα_s_ dissociation and β-arrestin recruitment to the β_1_AR via BRET, wherein the R3-truncated C11-G and C11-I analogs completely lost the antagonistic functions of C11 (Supplemental Figure 5, D–F), while the remaining analogs that varied in the R1 group remained functional antagonists to a similar or lesser extent than C11 (Supplemental Figure 5, D–F). Together, these data demonstrate that the identity of the R1 chemical group is largely exchangeable, while the R3 extended hydrocarbon chain is critical for its function and engagement with the receptor. These findings provide several mechanistic insights into the precise chemical features that mediate the activity of C11 and indicate that C11 is a superior PAM antagonist relative to a panel of structurally related molecules selected from the OpenDEL library. Lastly, C11-G, the R3-truncated form of C11, was revealed as a complete loss-of-function analog.

### C11 reduces basal contractility and suppresses the isoproterenol response in isolated WT cardiomyocytes.

**Figure 6:**
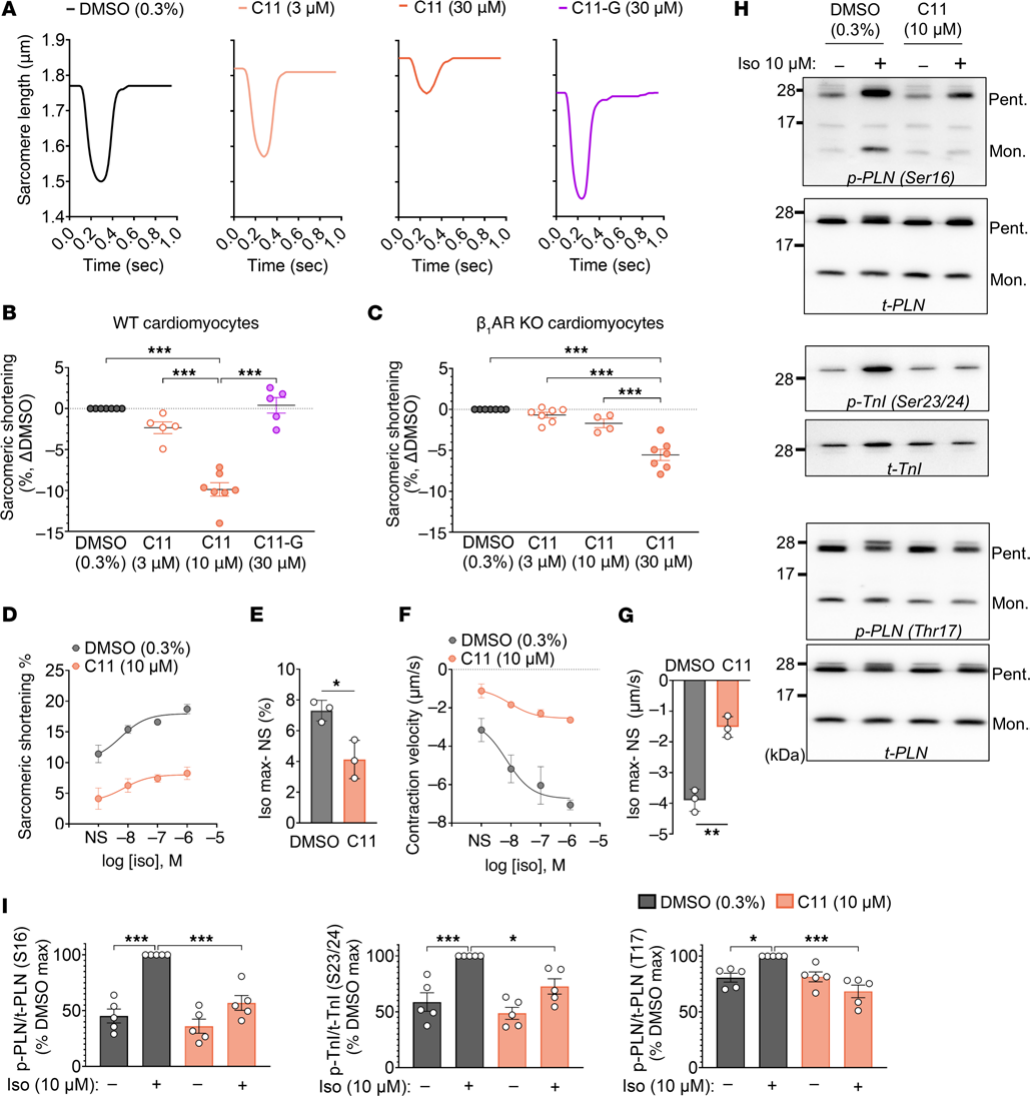
C11 reduces basal contractility and suppresses the isoproterenol response in isolated WT cardiomyocytes. (**A** and **B**) Representative unloaded shortening contractions (**A**) from isolated WT cardiomyocytes treated with increasing doses of C11 displayed a significant decrease in basal sarcomeric shortening in cardiomyocytes treated with 30 μM C11 compared with vehicle; treatment with loss-of-function C11 analog, C11-G, had no effect (**B**). (**C**) High doses of C11 (30 μM) significantly reduced basal sarcomeric shortening in β_1_AR^–/–^ cardiomyocytes, indicating C11-mediated reduction in contractility is partially nonselective. An intermediate dose of 10 μM was therefore selected for functional cardiomyocyte assays; 1-way ANOVA, ***P < 0.0002; data points represent biological replicates (*n* = 4–7 hearts, 7–10 cells per treatment per heart); absolute measurements of contractility parameters are in Supplemental Table 3. (**D**–**G**) Dose-dependent enhancement of sarcomeric shortening (**D**) and contraction velocity (**F**) stimulated by serial doses of isoproterenol (Iso) was significantly blunted (**E** and **G**) in isolated WT cardiomyocytes treated with 10 μM C11 compared with vehicle-treated cells. The magnitude of the isoproterenol-mediated increase is plotted as the difference between maximal isoproterenol dose (log[Iso] = –6 M) and nonstimulated (N.S.) condition (**E** and **G**); 2-tailed *t* test. *P < 0.05, **P < 0.01; data points represent mean ± SEM of *n* = 3 biological replicates (7–10 cells per treatment per heart). (**H** and **I**) Representative immunoblots (**H**) and corresponding densitometric quantifications (**I**) revealed significantly decreased levels of phosphorylated PLN (Ser16), TnI (Ser23/24), and PLN (Thr17) in C11-treated cardiomyocytes (10 μM) compared with vehicle control after isoproterenol stimulation. Phosphorylated proteins (pPLN and pTnI) are normalized to total levels (tPLN and tTnI). Statistical comparisons (1-way ANOVA) are shown between DMSO(vehicle) and DMSO(iso), C11(vehicle) and C11(iso), and between DMSO(iso) and C11(iso) groups only; *P < 0.03, **P < 0.002, ***P < 0.0002; data points represent biological replicates (*n* = 5 hearts, each performed in duplicate); Pent, pentamer; Mon, monomer.

After our comprehensive pharmacological and cellular characterizations, we investigated the impact of C11 on cardiac signaling and function in primary cardiomyocytes expressing endogenous levels of the β_1_AR. Ventricular cardiomyocytes isolated from adult WT mice were pretreated with DMSO or serial concentrations of C11 and paced at 1 Hz. Pretreatment with 30 μM C11 induced a significant reduction in basal fractional shortening compared with cells pretreated with DMSO (Figure 6, A and B, and Supplemental Table 3). To evaluate whether this inhibitory effect was specific to C11 and not a nonselective consequence of its high dosage (up to 30 μM), we employed the loss-of-function analog, C11-G, that does not modulate β_1_AR-mediated signaling (Supplemental Figure 5, A–F). Here, pretreatment with 30 μM C11-G did not affect basal contractility in WT cardiomyocytes compared with vehicle control, suggesting a direct effect of C11 on cardiomyocyte contractility (Figure 6, A and B, and Supplemental Table 3). To assess whether the C11-mediated reduction in basal contractility results from direct modulation of the β_1_AR, we pretreated ventricular cardiomyocytes isolated from β_1_AR^–/–^ mice with DMSO or serial concentrations of C11. Importantly, 30 μM C11 also elicited a significant reduction in basal contractility in β1AR^–/–^ cardiomyocytes (Figure 6C), albeit to a lesser extent than in WT cardiomyocytes (Figure 6, A and B), whereas pretreatment with 10 μM C11 did not alter basal contractility in β1AR^–/–^ cells. These data suggest that the C11-mediated suppression of basal contractility at high concentrations (i.e., 30 μM C11) is due to partial off-target effects, while 10 μM C11 shows little off-target effect. We therefore selected 10 μM C11 for subsequent functional and signaling assays in isolated cardiomyocytes.

To determine the effect of C11 on the isoproterenol-induced contractile response, we stimulated isolated WT cardiomyocytes with serial concentrations of isoproterenol in the presence or absence of 10 μM C11. In line with its ability to suppress Gα_s_ and β-arrestin signaling downstream of agonist-activated β_1_AR, C11 substantially suppressed the dose-dependent increase in fractional shortening and contractile kinetics mediated by isoproterenol (Figure 6, D–G). To corroborate this finding biochemically, we selected representative Ca^2+^ cycling or sarcomeric cellular effectors of β_1_AR activation, namely phospholamban (PLN) and troponin I (TnI), and assessed their phosphorylation status in isolated cardiomyocytes in the presence of isoproterenol via immunoblotting. Pretreating WT cardiomyocytes with 10 μM C11 robustly suppressed the isoproterenol-induced phosphorylation of PLN and TnI at the canonical PKA phosphorylation sites (pPLN Ser16 and pTnI Ser23/24) as well as Thr17 mediated by Ca^2+^/calmodulin protein kinase II (CaMKII) on PLN (Figure 6, H and I), providing further evidence of C11’s ability to block β_1_AR-mediated signaling in the heart.

### C11 restores regular contractile rhythm and suppresses spontaneous Ca^2+^ release in CSQ2^–/–^ cardiomyocytes.

**Figure 7:**
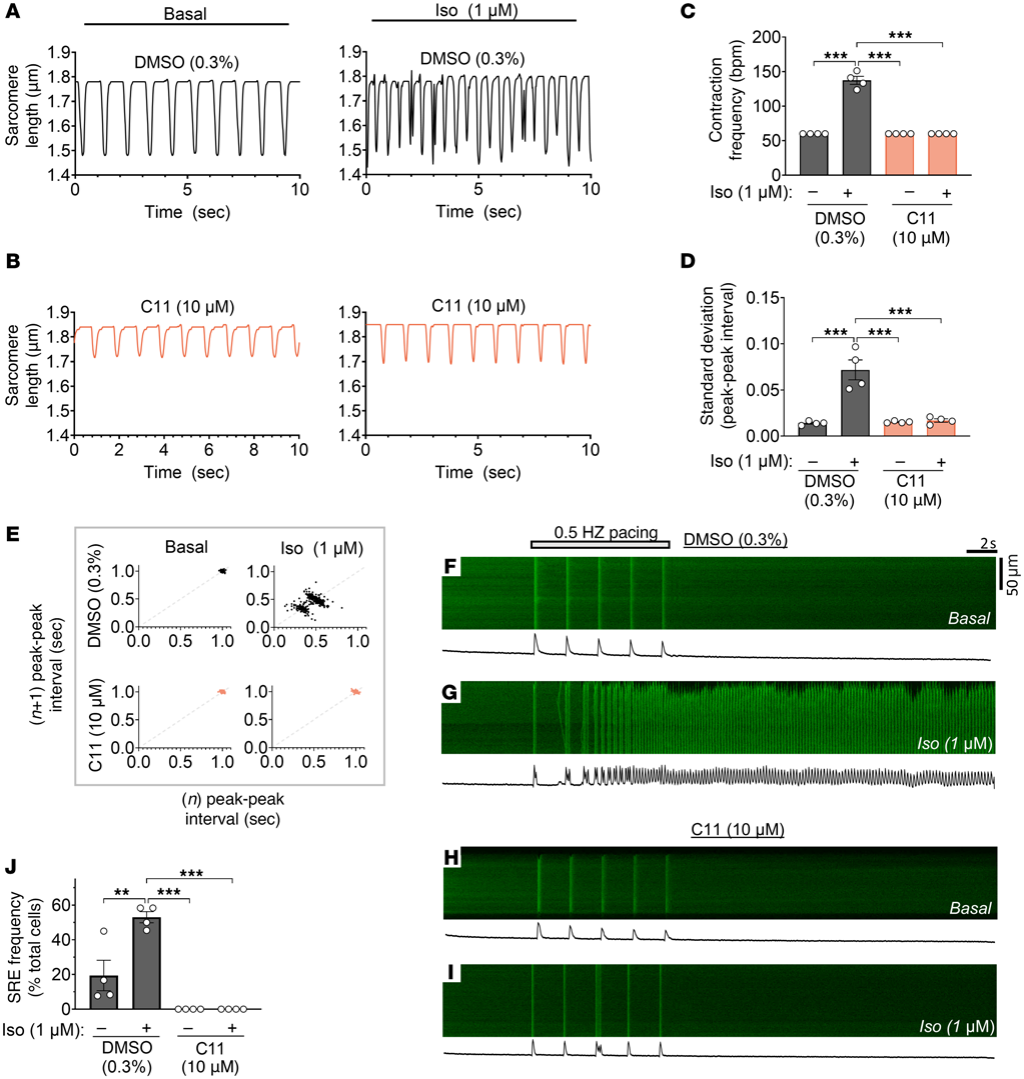
C11 restores regular contractile rhythm and suppresses spontaneous Ca^2+^ release events in cardiomyocytes isolated from CSQ2^–/–^ mice. (**A**–**D**) Representative unloaded shortening contractions (**A** and **B**) and corresponding quantifications (**C** and **D**) revealed frequent and irregular beats in vehicle-treated CSQ2^–/–^ cardiomyocytes stimulated with isoproterenol (Iso) during 1Hz pacing (i.e., 1-second interval), reminiscent of ventricular tachycardia. Isoproterenol-mediated spontaneous beating was completely attenuated in cells pretreated with 10 μM C11; data points represent biological replicates (*n* = 4 hearts, 7–10 cells per treatment per heart); 1-way ANOVA, ****P < 0.0002. (**E**) Poincaré plots depict increased variability in interval between consecutive cellular contractions in vehicle-treated CSQ2^–/–^ cardiomyocytes stimulated with isoproterenol but not in cells pretreated with 10 μM C11; data points represent peak-peak intervals of all biological replicates combined. (**F**–**J**) Spontaneous Ca^2+^ release events (SREs) were measured in quiescent ventricular cardiomyocytes following pacing at 0.5 Hz. Representative confocal line scans with associated fluorescent intensity profiles (**F**–**I**) and corresponding quantifications (**J**) revealed a robust increase in spontaneous Ca^2+^ release event frequency in vehicle-treated CSQ2^–/–^ cardiomyocytes after stimulation with isoproterenol (Iso, **G**) that was significantly attenuated in cells pretreated with 10 μM C11 (**I**); data points represent biological replicates (*n* = 4 hearts, 11–13 cells per treatment per heart); 1-way ANOVA, **P < 0.0013, ***P < 0.0002.

Given the robust inhibition of the isoproterenol response in cardiomyocytes pretreated with C11, we next evaluated its potential as a therapeutic molecule for CPVT utilizing cardiomyocytes from mice that are constitutively null for CSQ2 (26). Previous studies have demonstrated that CSQ2^–/–^ mice are highly susceptible to catecholamine-induced arrhythmia in the form of frequent premature ventricular contractions, increased heart rate variability, and increased diastolic Ca^2+^ leak due to spontaneous Ca^2+^ release events (26). Consistent with this, isolated ventricular cardiomyocytes from CSQ2^–/–^ mice developed an arrhythmic-like phenotype when stimulated with isoproterenol during 1 Hz pacing, evidenced by a robust increase in both the frequency of contractions and the variability in the time interval between consecutive beats that was reminiscent of the irregular contractile rhythms observed in vivo (Figure 7A) (26). Remarkably, pretreatment with 10 μM C11 completely attenuated isoproterenol-mediated spontaneous cellular beating and restored regular contractile frequency in CSQ2^–/–^ cardiomyocytes (Figure 7, A–E). To further corroborate the protective effect of C11 in CSQ2^–/–^ cardiomyocytes, we measured spontaneous Ca^2+^ release events elicited by isoproterenol immediately after termination of 0.5 Hz pacing. In line with the blockade of isoproterenol-induced spontaneous contractile activity in CSQ2^–/–^ cardiomyocytes (Figure 7, A–E), pretreatment with 10 μM C11 also prevented the development of spontaneous Ca^2+^ release events after isoproterenol stimulation (Figure 7, F–J). Of note, 10 μM C11 had no significant effect on the amplitude or kinetics of paced Ca^2+^ transients in CSQ2^–/–^ cardiomyocytes (Supplemental Figure 6, A–E), nor did it affect caffeine-induced Ca^2+^ release in WT cells (Supplemental Figure 7, A and B), indicating that C11 has no direct impact on baseline Ca^2+^ signaling.

### C11 suppresses exercise-induced ventricular tachycardia in vivo.

**Figure 8:**
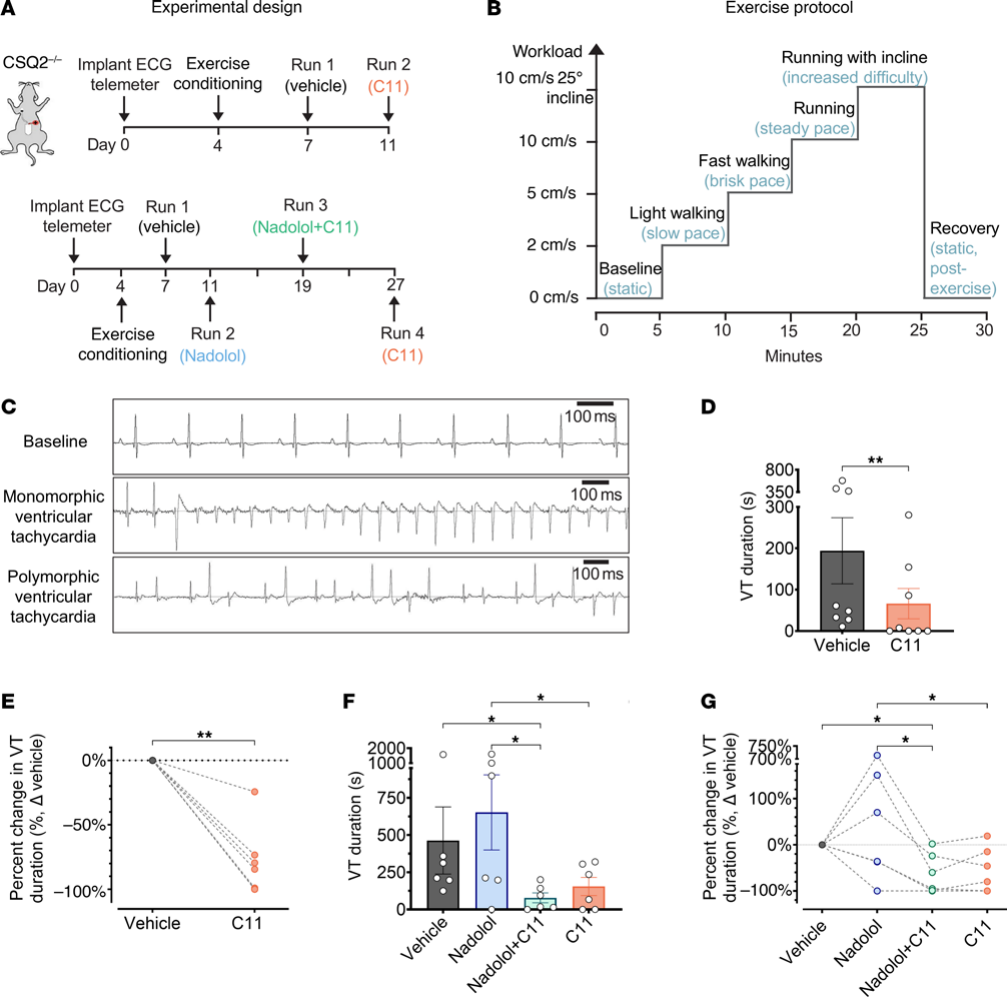
C11 suppresses the duration of exercise-induced ventricular tachycardia in vivo. (**A**) CSQ2^–/–^ mice were implanted with telemetry devices (day 0) and allowed to recover for 3 days before acclimation (days 4–6). Mice were pretreated with vehicle (day 7) or 10 mg/kg C11 (day 11) and subjected to a strenuous treadmill exercise protocol (upper panel). For combination experiments with nadolol (lower panel), mice had a 7-day washout between drug treatments. (**B**) Illustrative graph of 30-minute graded exercise protocol where workload (i.e., speed and incline) was incrementally increased every 5 minutes. Mice were placed on a single-lane treadmill with an adjustable incline (maximum 25°). Vehicle or drug solutions were administered intraperitoneally 45 minutes before exercise began. (**C**) Representative electrocardiograms obtained via continuous telemetric recording depict normal sinus rhythm at rest (baseline) and episodes of monomorphic and/or polymorphic ventricular tachycardia (VT) in CSQ2^–/–^ mice during physical exertion. (**D**) The total duration of VT was significantly reduced in CSQ2^–/–^ mice pretreated with C11 compared with when these same mice were pretreated with vehicle during exercise; data points represent biological replicates (*n* = 8 mice); Wilcoxon matched-pairs signed-rank test, **P < 0.01. (**E**) The percentage change in total VT duration after C11 treatment compared with vehicle was plotted for individual mice. (**F**) VT duration was measured in a separate cohort of CSQ2^–/–^ mice treated sequentially with vehicle solution (day 7), low-dose nadolol (0.01 mg/kg; day 11), combination of low-dose nadolol (0.01 mg/kg) and C11 (10 mg/kg; day 19), and C11 alone (10 mg/kg; day 27). The average VT duration was significantly lower when treated with C11 alone or in combination with low-dose nadolol compared with nadolol alone. (**G**) The percentage change in total VT duration after each treatment was plotted for individual mice; data points represent biological replicates (*n* = 6 mice); nonparametric repeated-measures 1-way ANOVA (Friedman’s test), *P < 0.05.

Intense exercise stress is the principal trigger for ventricular tachycardia (VT) and sudden cardiac death in individuals with CPVT. To investigate the therapeutic potential of C11 in a physiologically relevant setting, we recorded cardiac electrical activity via telemetry in conscious, nonanesthetized CSQ2^–/–^ mice subjected to graded treadmill exercises designed to mimic strenuous physical exertion in humans (Figure 8, A–C). To determine an appropriate dosing regimen for C11, we first confirmed that the compound is stable long-term (up to 72 hours) in vehicle solution at 37°C via high-performance liquid chromatography–mass spectrometry (Supplemental Figure 8A). We also obtained the pharmacokinetic profile of C11 after intraperitoneal injection (10 mg/kg) and determined that C11 was detectable in the plasma and heart at the highest level between 45 minutes and 1 hour after injection (Supplemental Figure 8B). After surgical implantation of the telemetry module in the peritoneal cavity, mice were allowed a 3-day recovery period followed by a 3-day acclimation phase during which they were conditioned to treadmill running (Figure 8A). Guided by our pharmacokinetic findings, treadmill-acclimated CSQ2^–/–^ mice were pretreated for 45 minutes with either vehicle solution or 10 mg/kg C11 and subjected to forced treadmill running where workload (i.e., speed and incline) was periodically increased over 30 minutes (Figure 8B). Individual mice were delivered alternating treatments of vehicle or C11 4 days apart (days 7 and 11, respectively; Figure 8A), and thus each mouse served as its own internal control. To ensure robust analysis of C11’s efficacy, mice lacking exercise-induced VT (i.e., less than 5 seconds) during vehicle treatment were not included for further study, limiting the assessment to animals with measurable baseline arrhythmogenic activity.

The total duration of sustained monomorphic and/or polymorphic VT defined as consecutive ectopic beats with unidirectional or bidirectional QRS waveforms, respectively (Figure 8C), was quantified for each animal during graded exercise. Remarkably, the total duration of VT encompassing both monomorphic and polymorphic forms was significantly attenuated in C11-treated CSQ2^–/–^ mice compared with when those same mice were pretreated with vehicle solution (Figure 8D). Given the variability in arrhythmic burden among vehicle-treated mice, we plotted the relative percentage change in total VT duration for individual mice (Figure 8E). This analysis revealed a median reduction in VT of approximately 50% after C11 treatment as compared with vehicle (Figure 8E). In addition to sustained episodes of VT, we assessed the incidence of premature ventricular contractions during the graded exercise test (Supplemental Figure 9A). Although most individual mice also developed a reduction in premature ventricular contractions after C11 treatment, this difference did not reach statistical significance (*P* = 0.195, Wilcoxon matched-pairs signed-rank test; Supplemental Figure 9, B and C). This may be attributed to the significant role catecholamines have in inducing and propagating sustained VT as compared with isolated premature ventricular contraction events.

We further tested the ability of C11 to suppress arrhythmic events in comparison with nadolol, an orthosteric beta blocker that remains the most efficacious and widely used pharmacological therapy for CPVT. To identify a dose of nadolol that elicited a mild but not complete reduction in heart rate in response to exercise, we sequentially treated individual WT mice with vehicle, 0.005 mg/kg, 0.01 mg/kg, and 0.1 mg/kg nadolol in a repeated-measures design (Supplemental Figure 10). Given that treatment with 0.1 mg/kg nadolol markedly suppressed the heart rate response to exercise and may represent a level of drug that is likely to cause symptomatic side effects, we selected 0.01 mg/kg as a low-dose nadolol condition to be tested in combination with C11. We subsequently delivered vehicle, low-dose nadolol (0.01 mg/kg), low-dose nadolol (0.01 mg/kg) in combination with C11 (10 mg/kg), or C11 alone (10 mg/kg) to individual CSQ2^–/–^ mice with a 7-day washout period between treatments (Figure 8A) and quantified VT duration during exercise. Treatment with C11in combination with low-dose nadolol significantly reduced the duration of exercise-induced VT compared with low-dose nadolol alone and to a similar extent as C11 alone (Figure 8, F and G).

Taken together, these results demonstrate the therapeutic capability of C11 in mitigating exercise-induced arrhythmias associated with the pathological overactivation of β_1_ARs in the heart and underscore its potential as a new class of drug to block the β_1_AR in disease states.

## Discussion

Herein, we report the discovery of the β_1_AR-selective allosteric modulator, C11 (and its analog molecules), via DEL screening and comprehensively interrogate its pharmacological properties, functional effects, and therapeutic applications in the heart. Our studies reveal that C11 binds to the β_1_AR with low-micromolar affinity and potentiates the binding of agonists and a subset of antagonists to the β_1_AR in a subtype-specific fashion. In contrast to its positive cooperativity with orthosteric agonists, C11 potently decreases agonist-activated β_1_AR signaling, suppresses the isoproterenol response in isolated cardiomyocytes, and largely prevents the development of spontaneous contractile and Ca^2+^release events in a model of CPVT. Most importantly, treatment with C11 significantly mitigates exercise-induced ventricular tachycardia in CPVT mice. C11 therefore represents a promising therapeutic molecule for the treatment of cardiac disease, illustrated herein in the context of CPVT.

CPVT is an inherited arrhythmic disorder characterized by the heightened susceptibility to catecholamine-induced polymorphic or bidirectional ventricular tachycardia initiated by exercise or emotional stress in the absence of structural heart disease (6). The genetic basis is commonly associated with mutations in proteins of the sarcoplasmic reticulum (SR) Ca^2+^ release complex (i.e., RyR2 or CSQ2), leading to SR Ca^2+^ overload, spontaneous Ca^2+^ release due to hyperactive RyR2, and arrhythmogenic Ca^2+^-induced delayed afterdepolarizations, potentially culminating in sudden cardiac death (6, 27). CPVT is a severe life-threatening disorder with mortality rates ranging from 30% to 50% in untreated patients by the age of 40 (28). Currently, beta blockers such as nadolol are the most efficacious therapy for CPVT and have been successful in decreasing arrhythmic risk and severity (7, 29). However, beta blockers are not completely effective given that recurrent arrhythmic events occur in up to 37.2% of treated patients, with 15.3% being near-fatal and 6.4% fatal during an 8-year follow-up period after beginning treatment (30). A major obstacle that remains is achieving a maximally tolerated dose of beta blocker due to adverse effects in vascular tissues (i.e., hypotension) where the β_2_AR is highly expressed (5, 8). Moreover, cardio-selective βAR antagonism would be especially optimal in the presence of respiratory disorders such as asthma or COPD. As a β_1_AR-selective allosteric modulator, C11 should maximize its inhibitory effects once all allosteric sites are occupied (i.e., the ceiling effect) and therefore has the potential to be used at a therapeutically effective dose with minimal off-target concerns (11). Beyond this, PAM antagonism may confer additional therapeutic benefits that are elaborated further below.

Although the present study demonstrates the therapeutic potential of C11 utilizing a model of CPVT where the primary pathological mechanism is directly related to β_1_AR activation, C11 could be applied as a treatment for a wide range of cardiac diseases (i.e., hypertrophic and dilated cardiomyopathy) or arrhythmic disorders (i.e., long QT syndrome, arrhythmogenic cardiomyopathy, atrial fibrillation) that are potentiated by excessive sympathetic stimulation and where beta blockers have been previously shown to be beneficial (7). It is also interesting to speculate that C11 could possibly modulate the activity of β_1_AR-activating autoantibodies that develop during chronic heart failure (31–33) and in conditions where enhanced sympathetic activity results in high synaptic catecholamine release, such as exertional angina and postural orthostatic tachycardia syndrome. An important consideration in the clinical use of C11 would be the probe dependence of its modulatory effects on distinct orthosteric beta blockers. Our studies showed that C11 potentiates the affinity of a subset of antagonists to the β_1_AR, such as the β-arrestin–biased ligand carvedilol, and suppresses carvedilol-mediated ERK activation. It remains to be determined whether C11 also diminishes the antagonist function of carvedilol on G protein signaling or whether C11 potentiates the inhibition. Further clarifying the pharmacological properties of C11 will be essential for assessing whether it could potentially be used in combination with orthosteric beta blockers, such as carvedilol, to enhance their efficacy. Our evaluation of the therapeutic effectiveness of C11 when used in combination with low-dose nadolol, however, implies that physiologically it may enhance the inhibitory function of certain orthosteric antagonists.

The unique pharmacological and functional profile exhibited by C11 shows features similar to a recently recognized subclass of allosteric modulators termed PAM antagonists that increase the binding affinity for receptors but decrease the functional efficacy of the agonist (23). There are several major advantages to this mechanism of signaling blockade relative to traditional orthosteric antagonists like beta blockers. Specifically, in contrast to orthosteric antagonists and even canonical NAMs that act competitively with the agonist by favoring an inactive receptor conformation, the potency and efficacy of a PAM antagonist is actually potentiated in the presence of increasing agonist concentration due to their positive cooperativity on agonist binding. Moreover, PAM antagonists such as C11 are further advantageous in that they have a higher propensity to bind agonist-bound receptors, thereby preferentially targeting preexisting pathological overactivation (23). Along with its selective effects on the β_1_AR, the pharmacological properties conferred by PAM antagonism underscore the potential advantageous therapeutic properties of C11 compared with canonical nonselective orthosteric beta blockers.

Although C11 has shown reciprocal effects on agonist binding affinity and functional efficacy, the precise inhibitory mechanism, binding site, and receptor conformation it promotes remain outstanding questions. To date, only several PAM antagonists are currently described, and their mechanism of action is largely elusive. The first reported PAM antagonist, ifenprodil, was discovered in 1996 as a neuroprotective modulator of the NMDA glutamate receptor ion channel (34). In the following decades, PAM antagonists targeting the free fatty acid 3 (FFA-3) receptor (35) and the cannabinoid 1 (CB1) receptor (36, 37) were identified. Previous studies utilizing site-directed fluorescent labeling characterizing the CB1 PAM antagonist Org27569 have reported that Org27569 blocks conformational changes associated with G protein binding, suggesting that it locks the CB1 receptor in an agonist-bound, nonsignaling, early-activation intermediate state (38). Alternative hypotheses regarding the mechanism of PAM antagonists include stabilizing a nonsignaling quaternary complex composed of receptor, agonist, modulator, and transducer, or perhaps steric blockade of transducer binding. Signaling bias should also be considered, as some PAM antagonists could have selective effects on specific pathways like SBI-553, a β-arrestin–biased PAM of the neurotensin receptor 1 (NTSR1) that selectively suppresses Gαq signaling while potentiating β-arrestin signaling through promoting a β-arrestin–selective receptor conformation (39, 40). We have generated some preliminary structure-activity insights into C11 via characterizing a panel of analogs, but future biophysical and structural studies will be required to precisely determine the binding location and mechanism of action of C11.

Through a DEL screen utilizing the OpenDEL small-molecule library (HitGen Inc.) comprising over 1 billion unique compounds, we discovered a subtype-selective PAM-antagonist allosteric modulator of the β_1_AR. Given its pharmacological and functional profile and demonstrated efficacy in suppressing pathological contractile and electrophysiological events in experimental CPVT, C11 is a promising therapeutic candidate that holds great potential to be developed as a drug for a wide range of heart diseases with pathological susceptibility to high-catecholamine states.

## Methods

### Sex as a biological variable.

Experiments evaluating the therapeutic potential of C11 in suppressing exercise-induced VT were conducted using male CSQ2^–/–^ mice. Female CSQ2^–/–^ mice did not exhibit measurable baseline arrhythmogenic activity during exercise. Therefore, female CSQ2^–/–^ mice were not included in the final analysis. Given these apparent sex differences inherent to the CSQ2^–/–^ phenotype, the efficacy of C11 in vivo was evaluated using male mice only.

### Radioligand competition binding.

Radioligand competition binding experiments were performed as previously described (22). Briefly, purified β_1_AR, β_1_V_2_Rpp, or β_2_AR nanodiscs generated as described in the Supplemental Methods were incubated with 60 pM of the radiolabeled orthosteric antagonist, [I^125^]-cyanopindolol (I^125^CYP; 2,200 Ci/mmol, PerkinElmer), and serial concentrations of unlabeled orthosteric ligand in binding buffer (20 mM HEPES pH 7.4, 100 mM NaCl) supplemented with 0.1% BSA and 1 mM ascorbic acid at room temperature for 2 hours to reach equilibrium. Nonspecific binding was evaluated in the presence of 20 μM propranolol. To validate nanodisc preparations, serial concentrations of purified heterotrimeric G_s_ (10–320 nM) or β-arrestin1-mc (0.1–1 μM) were included in the binding reaction with β_1_AR or β_1_V_2_Rpp nanodiscs, respectively, to confirm transducer cooperativity (Supplemental Figure 1, C and D). To evaluate the effect of allosteric modulators on orthosteric ligand binding to βARs, reactions included the indicated concentration of allosteric compound (0.05–30 μM) or an equivalent volume of vehicle (0.19% DMSO). Equilibrated binding reactions were harvested by rapid filtration onto 0.3% polyethyleneimine-soaked grade GF/B glass fiber filter paper (Brandel) and washed extensively with ice-cold binding buffer. I^125^-CYP was detected with the WIZARD^2^ 2-detector gamma counter (PerkinElmer), and the raw cpm was normalized to the percentage of maximal I^125^-CYP binding. The mean ± SEM of at least 3 independent experiments performed in duplicate were plotted in GraphPad Prism and fit to a 1-site binding model to retrieve IC_50_. C11-mediated log shifts in the nonlinear fit are presented as ΔIC_50_ (Vehicle – C11), and statistical analysis was performed using a paired 2-tailed *t* test of raw IC_50_ values (Supplemental Table 1). Statistical comparisons of the ΔIC_50_ between C11 and its analogs (C11A-I) were conducted by 1-way ANOVA with Dunnett’s post hoc test (GraphPad Prism).

### G protein dissociation assay (TRUPATH).

G protein dissociation was evaluated by the BRET-based TRUPATH assay as originally described in Olsen et al. (17) with minor modifications. Briefly, 2.25 × 10^6^ HEK293T cells maintained in growth media were seeded in a 10 cm dish and incubated overnight. On the next day, cells were transfected with 0.75 μg of human FLAG-β_1_AR (or FLAG-β_2_AR), with Gα_s_S-RLuc8, Gβ3, and Gγ9-GFP at a 1:1:1:1 DNA ratio. For AT1R counter-assays, 0.75 μg of human FLAG-AT1R (41) was transfected along with 0.75 μg of Gα_q_-RLuc8, Gβ3, and Gγ9-GFP. After 24 hours, cells were trypsinized, resuspended in low-serum medium (1× MEM without phenol red, supplemented with 2% FBS, 1% HEPES, 1% anti-anti, 1% glutamine, and 1% penicillin-streptomycin), and replated in a white, clear-bottom 96-well assay plate at a density of 100,000 cells/well for overnight incubation. Prior to experimentation, low-serum media was aspirated and replaced with 1× assay buffer (HBSS, supplemented with 20 mM HEPES) and incubated for 10 minutes at 37°C. Cells were pretreated with vehicle (0.19% DMSO) or 30 μM C11 prepared in 1× assay buffer for 20 minutes at 37°C. To assess β_1_AR-mediated G protein dissociation, 100 nM ICI-118,551 was included during pretreatment to block activation of endogenous β_2_ARs. Cells were then stimulated with serial concentrations of isoproterenol for 10 minutes at 37°C. BRET emission ratios (GFP/RLucII) were measured immediately after the addition of 5 μM coelenterazine 400a (Nanolight Technology) on a BioTek Neo2 microplate reader (Agilent Technologies) using the 410 nm (donor) and 515 nm (acceptor) filter pair. At least 3 independent experiments performed in duplicate were fitted to a log(agonist) versus response (3 parameter) model in GraphPad Prism and baseline subtracted to assess net BRET ratio. Statistical analysis of the nonlinear curve fit (E_max_) was evaluated by 2-tailed *t* test.

### GloSensor assay.

The GloSensor assay (Promega) was performed as previously described with minor modifications (22). Briefly, 2.25 × 10^6^ HEK293T cells maintained in growth media were seeded in a 10 cm plate and transfected 24 hours later with 25 ng of human FLAG-β_1_AR and 6 μg of the GloSensor cAMP biosensor plasmid (Promega). On the following day, cells were replated into a white, clear-bottom 96-well plate at a density of 50,000 cells/well in low-serum medium (1×MEM without phenol red, supplemented with 2% FBS, 1% HEPES, 1% anti-anti, 1% glutamine, and 1% penicillin-streptomycin) and incubated overnight. Prior to experimentation, low-serum media was aspirated and replaced with the GloSensor reagent (2 mM; Promega) prepared in 1× assay buffer (HBSS with 20 mM HEPES) and incubated at room temperature for 1.5 hours. Cells were pretreated with vehicle (0.19% DMSO) or 30 μM C11 prepared in 1× assay buffer for 20 minutes at room temperature, along with 100 nM ICI-118,551 to block activation of endogenous β_2_ARs. Cells were then stimulated with serial concentrations of isoproterenol for 5 minutes and luminescent values were recorded using the BioTek Neo2 microplate reader (Agilent Technologies). The mean ± SEM of at least 3 experimental replicates performed in duplicate were fit to a log(agonist) versus response (3 parameter) model in GraphPad Prism and normalized to the percentage of vehicle maximum. Statistical analysis of the nonlinear curve fit (E_max_) was evaluated by 2-tailed *t* test.

### BRET-based β-arrestin recruitment and receptor internalization assays.

Evaluation of β-arrestin recruitment and internalization was performed using BRET-based biosensors. One day prior to transfection, 2.25 × 10^6^ HEK293T cells maintained in growth media were seeded in a 10 cm dish. For β-arrestin recruitment assays, cells were cotransfected with 2 μg FLAG-β_1_V_2_R-RLucII and 1 μg β-arrestin2-eGFP (Addgene plasmid 35411; ref. 42); β-arrestin internalization was evaluated via cotransfection of 2 μg FLAG-β_1_V_2_R (or FLAG-β_2_V_2_R or FLAG-AT1R for counter-assays), with 1.5 μg β-arrestin2-RLucII, and 2.5 μg rGFP-FYVE (18). After 24 hours, cells were trypsinized, resuspended in low-serum medium (1× MEM without phenol red, supplemented with 2% FBS, 1% HEPES, 1% anti-anti, 1% glutamine, and 1% penicillin-streptomycin), and replated at a density of 100,000 cells/well in a white, clear-bottom 96-well plate for overnight incubation. Prior to experimentation, low-serum media was aspirated and replaced with 1× assay buffer (HBSS with 20 mM HEPES) and incubated for 10 minutes at 37°C. Cells were pretreated with vehicle (0.19% DMSO) or 30 μM C11 prepared in 1× assay buffer for 20 minutes at 37°C. To evaluate BRET responses mediated by β_1_AR only, 100 nM ICI-118,551 was included during pretreatment to block activation of endogenous β_2_ARs. Cells were then stimulated with serial concentrations of isoproterenol for 20 minutes (recruitment) or 25 minutes (internalization) at 37°C. BRET emission ratios (GFP/RLucII) were measured immediately after the addition of 5 μM coelenterazine 400a (Nanolight Technology) on a BioTek Neo2 microplate reader (Agilent Technologies) using the 410 nm (donor) and 515 nm (acceptor) filter pair. At least 3 independent experiments performed in duplicate were fitted to a log(agonist) versus response (3 parameter) model in GraphPad Prism and baseline subtracted to assess net BRET ratio. Statistical analysis of the nonlinear curve fit (E_max_) was evaluated by 2-tailed *t* test.

### Cardiomyocyte isolation.

Adult mouse ventricular cardiomyocytes were isolated using standard Langendorff perfusion procedures as previously described (43) from homozygous 12–16-week-old C57BL/6J WT, β_1_AR-KO (β_1_AR^–/–^) (44, 45), or calsequestrin 2 (CSQ2^–/–^) (26) mice. Mice were injected intraperitoneally with 200 U of heparin and anesthetized under 3% isoflurane. Dissected whole hearts were placed immediately into perfusion buffer (120 mM NaCl, 14.8 mM KCl, 0.6 mM KH_2_PO_4_, 0.6 mM Na_2_HPO_4_, 1.2 mM MgSO_4_ × 7H_2_O, 10 mM HEPES, 4.6 mM NaHCO_3_, 30 mM taurine, and 5.6 mM glucose, pH 7.3) and cannulated through the aorta. Hearts were perfused in retrograde for 3 minutes with oxygenated perfusion buffer and then for approximately 8 minutes with digestion buffer containing 2.4 mg/mL collagenase (Worthington) at 37°C. To terminate enzymatic digestion, ventricular tissues were transferred to perfusion buffer containing 10% calf serum with 12.5 μM CaCl_2_. Myocytes were dissociated by trituration and gradually brought to physiological Ca^2+^ (1.2 mM).

### Contractility measurements.

Cardiomyocytes isolated as described above were pretreated with DMSO (0.3%) or C11 (3–30 μM) for 20 minutes. After pretreatment, cells were stimulated with isoproterenol (0.01–1 μM) or left untreated (basal condition) and plated immediately in an FHD rotational cell chamber (Ionoptix) mounted on a Nikon Eclipse TE300 inverted microscope (40× 0.9 NA objective, MRF00400, Nikon). Myocytes were paced at 1Hz (20 V, MyoPacer, Ionoptix), and sarcomere length was recorded with IonWizard 7.2 using the MyoCam-S camera (Ionoptix). Ten consecutive contractions per cell were averaged for quantification of contractile magnitude and kinetics (IonWizard 7.2). At least 7–10 cells were quantified per experimental condition and averaged. Time intervals between consecutive contractions (i.e., peak-peak intervals) were measured during a representative 5-second recording in each cell. Interval variability plots were generated by plotting the peak-peak interval (*n*) against the peak-peak interval of the subsequent cellular contraction (*n* + 1) for all cells in a particular treatment group. Only the myocytes that exhibited proper morphology (i.e., rod-shaped and striated) and were responsive to electrical stimulation were utilized for experimentation. Statistical comparisons between conditions were evaluated by 1-way ANOVA with Tukey’s post hoc test.

### Phospho-PLN and phospho-TnI assay.

Cardiomyocytes isolated as described above were pretreated with DMSO (0.3%) or 10 μM C11 for 20 minutes at room temperature prior to stimulation with 10 μM isoproterenol for 15 minutes while rotating. Cells were pelleted via centrifugation and homogenized in a 1:1 mixture of urea-thiourea lysis buffer (8 M urea, 2 M thiourea, 3% SDS, 0.05 M tris-HCl, 0.03% bromophenol blue, 0.075 M DTT, pH 6.8) supplemented with Halt protease and phosphatase inhibitor cocktail (78440, Thermo Fisher Scientific) and 50% glycerol at 60°C as described in Grogan et al. (46). Lysates were separated on a 15% SDS-polyacrylamide gel, and membranes were probed with the following primary antibodies: rabbit polyclonal antibodies targeting pPLN Ser16 (1:1,000, 07-052, MilliporeSigma), pTnI Ser23/24 (1:1,000, 4004S, Cell Signaling Technology), pPLN Thr17 (1:1,000, Arigo Biolaboratories), and TnI (1:1,000, 4002S, Cell Signaling Technology), or mouse monoclonal antibodies to PLN (ab2865, Abcam) with HRP-conjugated secondary antibodies (1:3,000; donkey anti-rabbit IgG, NA934V, and sheep anti-mouse IgG, NA931VS, Cytiva). Densitometric analysis of phospho-PLN and phospho-TnI was performed with ImageJ (NIH) and normalized to total PLN or TnI, respectively. Each experimental condition was performed in duplicate and averaged per biological replicate. Statistical analysis was performed with 1-way ANOVA and Tukey’s post hoc test.

### Cardiomyocyte Ca^2+^ imaging.

To conduct live cell Ca^2+^ imaging, ventricular myocytes isolated as described above were loaded with 10 μM CAL-520 (ab171868, Abcam) for 1 hour in 1× Ca^2+^-free Tyrode solution. Cells were then incubated with a 1:1 solution of RPMI/B27 plus RPMI 1640 (11875199, Thermo Fisher Scientific) with 2% B27 with insulin (17504044, Life Technologies) and 1× Tyrode with 1.8 mM CaCl_2_ for 20 minutes prior to imaging. Cells were pretreated with DMSO (0.3%) or 10 μM C11 for 20 minutes and plated onto coverslips coated with 200 μg/mL laminin (L2020, Sigma-Aldrich). Line scans were acquired on a Zeiss laser scanning confocal 510 meta microscope at 0.1 μm per pixel along the longitudinal axis of cardiomyocytes. Cells were paced at 0.5 Hz with an IonOptix MyoPacer field stimulator (IonOptix) for 10 seconds with a total imaging time of 34 seconds per cell. Fiji ImageJ v.1.53c (NIH) was used to analyze Ca^2+^ transients. Cells were scored as having sustained spontaneous calcium release events (SREs) if there were greater than 10 SREs less than 500 ms apart. For caffeine-induced Ca^2+^ transients, cells were paced at 0.5 Hz for 10 seconds and then treated with 10 mM caffeine (10 seconds after termination of pacing) on a Zeiss spinning disk Axio Observer.Z1 (40×, 215 ms intervals). All Ca^2+^ imaging experiments were performed at room temperature.

### Mouse graded treadmill exercise.

Exercise experiments were performed with both young (8–12 weeks) and old (12 months) male CSQ2^–/–^ mice. Genotypes were confirmed via PCR using previously described protocols (26, 44, 45, 47). CSQ2^–/–^ mice were implanted with wireless ECG telemetry units (model ETA-F10, DSI-Harvard Bioscience Inc.) and allowed to recover for 3 days. Female mice implanted with ECG telemeters were not used for these experiments since they did not show exercise-induced VT on repeated exercise testing. Pre-exercise conditioning involved acclimating mice to the rodent treadmill (PanLab LE8708TS, Harvard Apparatus) for 30 minutes/day on 3 consecutive days. On the first day of the acclimation period, mice were placed on a stationary treadmill belt and allowed to explore the treadmill chamber for 30 minutes. On the subsequent 2 days of acclimation, the treadmill speed was set to 2 cm/s, 5 cm/s, and 10 cm/s at a 25° incline for 10 minutes at each speed. Acclimated mice were then subjected to a rigorous graded exercise protocol following a 45-minute pretreatment with vehicle solution (10% dimethylacetamide, 40% PEG 300, and 2% Tween 80 in 48% saline) delivered intraperitoneally. The graded exercise protocol consisted of 5 stages where workload was incrementally increased every 5 minutes: 0 cm/s (baseline), 2 cm/s (light walking), 5 cm/s (fast walking), 10 cm/s (running), and 10 cm/s with a 25° incline (running with increased difficulty), followed by a 5-minute recovery period at 0 cm/s. After 3 days of washout, mice were again subjected to the same graded exercise protocol following a 45-minute pretreatment with 10 mg/kg C11 prepared in vehicle solution delivered intraperitoneally (Figure 8A).

For the nadolol dose-finding experiments, the graded treadmill stress test was performed repeatedly on individual WT mice with a 7-day washout period between sessions to ensure complete drug clearance before subsequent experiments. Heart rate was recorded during the pre-running baseline period (while positioned on the treadmill), as well as during walking (2 cm/s) and running (5 cm/s). For evaluation of low-dose nadolol prepared either alone or in combination with C11, drugs were administered intraperitoneally to CSQ2^–/–^ mice with a 7-day washout period in between (Figure 8A).

To ensure the analysis was focused on CSQ2^–/–^ mice with measurable baseline arrhythmogenic activity, mice lacking exercise-induced VT (i.e., less than 5 seconds) during the vehicle-treated exercise run were not included in the study. Negative reinforcement with the shock grid set to 0.4 mV was used to encourage running. The treadmill experiments were conducted and reviewed with continuous data acquisition using Ponemah v6.6 software (DSI-Harvard Bioscience, Inc). The treadmill was disinfected with 70% ethanol after each session. Monomorphic ventricular tachycardia was defined as 4 or more consecutive ventricular complexes with a consistent intrinsic shape, peak-to-peak interval, and amplitude. Polymorphic ventricular tachycardia was defined as ventricular complexes with progressive variations in intrinsic shape, peak-to-peak interval, or amplitude (48). Statistical comparisons of the duration of ventricular tachycardia and the total number of premature ventricular contractions were assessed by Wilcoxon matched-pairs signed-rank test or nonparametric repeated-measures 1-way ANOVA (Friedman’s test).

### Statistics.

Statistical significance was determined by 2-tailed *t* test or 1-way ANOVA with Tukey’s post hoc test for multiple comparisons (GraphPad Prism) unless indicated otherwise. Comparisons between the IC_50_ of competition binding curves or the nonlinear fit (E_max_) of in vitro signaling assays were performed using an *F* test (GraphPad Prism). Data are shown as mean ± SEM. Sample sizes are indicated in the corresponding figure legends. *P* values of less than 0.05 were considered statistically significant.

### Study approval.

All animal procedures were performed in accordance with NIH guidelines (*Guide for the Care and Use of Laboratory Animals*, National Academies Press, 2011) and were approved by the IACUC at Duke University Medical Center.

### Data availability.

Data underlying each graph are reported in the Supporting Data Values file. Additional descriptions of the materials and methods, including sample sizes and statistical analyses, are included in the supplemental materials and corresponding figure legends.

## Author contributions

AG, SA, RJL, and HAR conceptualized the study. AG, RMP, SA, HJ, AJ, DS, CY, QC, JL, JW, and AWK developed the methodology. AG, RMP, HJ, AJ, DS, CY, JW, and AWK performed experiments. AG, RMP, HJ, AJ, DS, CY, QC, JL, JW, and AWK analyzed data. JS, RJL, and HAR provided resources and/or reagents. HAR acquired funding. SA, DII, AS, APL, RJL, and HAR supervised the study. AG wrote the original draft of the manuscript. All authors reviewed, edited, and approved the manuscript for publication.

## Funding support

This work is the result of NIH funding, in whole or in part, and is subject to the NIH Public Access Policy. Through acceptance of this federal funding, the NIH has been given a right to make the work publicly available in PubMed Central.

- NIH and National Heart, Lung, and Blood Institute grants HL056687 to HAR and HL16037 to RJL.
- The Edna and Fred L. Mandel, Jr. Foundation to HAR.
- The John Taylor Babbit Foundation and the Hartwell Foundation to APL.
- National Cancer Institute Comprehensive Cancer Center Core grant P30CA014236 to the Duke Pharmacokinetics and Pharmacodynamics Shared Resource Core.
- RJL is an investigator with the Howard Hughes Medical Institute.

## Supplementary Material

Supplemental data

Unedited blot and gel images

Supporting data values

## Version 1 10/16/2025

In-Press Preview

## Version 2 12/15/2025

Electronic publication
