# Supplementary material for: A positive allosteric modulator of the β1AR with antagonist activity for catecholaminergic polymorphic ventricular tachycardia
Source: J Clin Invest. 2025 Oct 16;135(24):e190252. doi: 10.1172/JCI190252 (PMC12700548; doi:10.1172/JCI190252)
Supplement: Supplemental data [file jci-135-190252-s155.pdf]

Supplementary Materials and Methods:

Materials

The following orthosteric ligands for βARs or AT1R were purchased commercially: isoproterenol hydrochloride, epinephrine hydrochloride, norepinephrine bitartrate, carvedilol, metoprolol tartrate, ICI 118,551 hydrochloride, atenolol, angiotensin-II (Sigma-Aldrich, St. Louis, MO), dobutamine hydrochloride, carazolol (Cayman Chemical, Ann Arbor, MI), bucindolol (Santa Cruz Biotechnology, Dallas, TX), and alprenolol hydrochloride (Tocris Bioscience, Minneapolis, MN). BI-167107 was used as a high affinity agonist of the β_1AR and β_2AR (1). Heterotrimeric G_s (2), minimal cysteine β-arrestin1 truncated at amino acid 393 (β-arrestin1-mc) (3, 4), nanobody 35 (Nb35) (2), and antibody fragment 30 (Fab30) (5) were expressed and purified as previously described. Nanobody 25 (Nb25) was isolated and purified as previously described for Nb32 (6). The DNA-encoded small molecule library OpenDEL™ was synthesized by HitGen Inc. (Chengdu, China) through a ‘split-and-pool’ approach. Off-DNA synthesis of hit compounds was performed and validated with HPLC/MS (>90% purity) by HitGen Inc.

FLAG-β_1V_2R and FLAG-β_1V_2R-RLucII constructs were generated using HiFi DNA assembly (New England Biolabs, Ipswich, MA) according to the manufacturer’s guidelines. Fragment inserts of human β_1AR truncated at residue G413 (β_1AR_G413), the C-terminal tail of human vasopressin 2 receptor (V_2R) encompassing amino acids 343-371 (V_2R_343-371), and V_2R_343-371 conjugated to RLucII were amplified by polymerase chain reaction (PCR) from pcDNA3 FLAG-β_1AR (Addgene plasmid 14698; (7)) or V_2R-RLucII (a gift from Dr. Sudar Rajagopal’s lab, Duke University) using primers listed in Supplemental Table 2. Gel-purified fragments were incubated with HiFi Master Mix (New England Biolabs) and inserted into a

linearized pcDNA3 empty vector (Addgene plasmid 10792). Plasmids were transformed into TOP10 *E. coli* (Thermo Fisher Scientific, Waltham, MA) and validated by DNA sequencing.

Cell culture

Human embryonic kidney (HEK) 293T cells were maintained in Minimum Essential Medium (MEM) supplemented with 10% fetal bovine serum (FBS) and 1% penicillin/streptomycin (P/S) in a humidified tissue culture incubator at 37 ˚C and 5% CO_2. For bioluminescence resonance energy transfer (BRET) and GloSensor™ (Promega, Madison, WI) cellular signaling assays, HEK293T cells were transiently transfected using Lipofectamine™ 3000 (Thermo Fischer Scientific, Waltham, MA) according to the manufacturer's standard protocol. Expi293T™ suspension cells stably expressing FLAG-β_1AR (8), FLAG-β_1AR_G413, or FLAG-β_2AR (9) were cultured in Expi293 Expression Medium (Invitrogen) with 10 µg/mL blasticidin and 10 µg/mL zeocin in a humidified tissue culture incubator maintained at 37 ˚C and 8% CO_2 under constant shaking.

Receptor purification and generation of receptor nanodiscs

Human FLAG-β_1AR and FLAG-β_2AR were expressed, purified, and reconstituted in high-density lipoprotein (HDL) particles to generate nanodiscs as previously described (4, 8). Biotinylation of the nanodisc membrane scaffold protein D1E3 (MSPD1E3) was utilized to facilitate immobilization to neutravidin beads. Chimeric β_1AR containing the phosphorylated C-terminal tail of the vasopressin 2 receptor (V_2R) was generated as previously described for the β_2V_2Rpp (4). Briefly, the sortase recognition sequence (LPETGHH) was inserted into the C-terminus of human FLAG-β_1AR after amino acid G413 to generate FLAG-β_1AR_G413 (SFig. 1A-

B). Following expression and detergent solubilization, FLAG-β_1AR_G413 was ligated to the synthetic phospho-peptide corresponding to the C-terminal tail of the V_2R (GGG-V_2Rpp) via incubation with sortase to generate FLAG-β_1V_2Rpp (SFig. 1B) (4). Prior to screening, reconstituted FLAG-β_1AR and FLAG-β_1V_2Rpp nanodiscs were validated by radioligand binding to ensure nanodiscs contained functional receptors (SFig. 1C-D).

Validation of the screening protocol

To measure the immobilization efficiency of purified β_1AR or β_1V_2Rpp biotinylated nanodiscs, a fixed quantity of pre-washed high capacity neutravidin beads (Pierce) were incubated with increasing concentrations of nanodisc in binding buffer (20 mM HEPES pH 7.4, 100 mM NaCl) at room temperature for 1 hour while rotating. After collecting flow through, the nanodisc-coated beads were washed three times with binding buffer and bound protein was eluted via boiling at 95 ˚C. Samples were loaded onto 10% SDS-polyacrylamide gels for analysis by Coomassie Blue and a 1:1 ratio of receptor nanodisc (µg) to bead slurry (µL) was deemed optimal given minimal loss of nanodisc in the flow through (SFig. 2A).

To confirm the integrity of the G protein and β-arrestin complexes throughout the screening procedure, β_1AR or β_1V_2Rpp nanodiscs were immobilized as described above to pre-washed high-capacity neutravidin beads along with 20 µM of the high-affinity agonist, BI-167107 (BI), and a 1.2 molar excess of heterotrimeric Gs or β-arrestin1-mc, respectively. To enhance complex stability, β_1AR/Gs complexes were supplemented with Nb35 (2.5 molar excess with respect to β_1AR) and 0.05 U/mL apyrase, while the β_1V_2Rpp/β-arrestin1 complex was further stabilized with Nb25 and Fab30 (2.5 or 1.7 molar excess relative to β_1V_2Rpp, respectively). Following complex

formation, the flow through was collected and the beads were washed three times with ice-cold binding buffer supplemented with 10 µM BI. To simulate incubation with DNA-encoded molecules, 1mg/mL salmon sperm DNA (Ambion) was applied to neutravidin-immobilized β_1AR/Gs and β_1V_2Rpp/β-arrestin1 complexes in binding buffer containing 20 µM BI for 1 hour while agitating (1150 rpm). The secondary flow through was collected, bound protein was eluted via boiling at 95 ˚C, and samples were loaded onto 10% SDS-polyacrylamide gels for analysis by Coomassie Blue to ensure the stability of receptor-transducer complexes throughout the selection protocol (SFig. 2B-C).

To validate the retention of small molecule ligands to the β_1AR or β_1V_2Rpp nanodiscs during screening, 5 nM of the radiolabeled orthosteric antagonist [H^3]-dihydroalprenolol (H^3-DHA, 105 Ci/mmol, PerkinElmer) was incubated with neutravidin-immobilized nanodiscs and washed three times with ice-cold binding buffer. To elute, beads were resuspended in 1.5% Fos-choline (Anatrace) in water and incubated at 37 ˚C (15 min) then 95 ˚C (15 min) while agitating (1150 rpm). The elution procedure was repeated a second time, combined, and applied to 5 mL scintillation fluid (LefkoFluor) for overnight incubation. H^3-DHA counts were obtained with a TriCarb 2800TR liquid scintillation counter (PerkinElmer). Empty nanodiscs were utilized as a control to assess non-specific binding (SFig. 2D).

DNA-encoded small molecule library screening

Dried aliquots of OpenDEL™ were resuspended in 50 µL water and incubated overnight at 4 ˚C to dissolve fully. Approximately 60 µg (~0.5 nmol) of β_1AR or β_1V_2Rpp nanodiscs were immobilized to pre-washed high capacity neutravidin beads along with transducer proteins (if

applicable), 20 μM BI (if applicable) and conformation stabilizing reagents as described above in 500 μL of binding buffer. Complexes were incubated for one hour at room temperature while rotating and washed three times with 1 mL of ice-cold binding buffer containing 10 μM BI. Prior to library incubation, 1 μL (2%) of dissolved OpenDEL™ was set aside for qPCR as input. The washed nanodisc-coated beads were resuspended in OpenDEL™, further diluted to 100 μL in ice-cold binding buffer supplemented with 1 mg/mL salmon sperm DNA and 20 μM BI, and incubated for 1 hour at room temperature while agitating. To remove unbound molecules, beads were washed three times with 500 μL ice-cold binding buffer containing 1 mg/mL salmon sperm DNA and 20 μM BI. Samples were eluted twice in 53 μL water containing 1.5% Fos-choline while agitating at 37 ˚C (15 min) then 95 ˚C (15 min). The combined elution was applied to the QIAquick Nucleotide Removal Kit (Qiagen, Hilden, Germany) and eluted in 70 μL water to isolate DNA-encoded molecules. Following DNA purification, 1.4 μL (2%) was set aside for qPCR analysis and the remaining elution was diluted to 100 μL in binding buffer containing 1 mg/mL salmon sperm DNA and 20 μM BI to apply as library input for a second round of affinity selection with freshly immobilized protein complexes. Following a second round of selection and purification of DNA-encoded molecules, samples were PCR amplified and subjected to next-generation DNA sequencing (HitGen Inc.) to decode binders. Of note, BI was omitted from wash buffers in the apo-β_1AR and empty nanodisc conditions. All solutions were prepared in DNase/RNase free UltraPure distilled water (Invitrogen, Waltham, MA) and all centrifugation steps were performed at 3000 xg for 1 min to pellet beads.

Determining library decay by qPCR

Aliquots of library input and elution collected as described above were diluted in qPCR sample buffer (10 mM Tris pH 8, 0.05% tween-20) and amplified along a standard curve of OpenDEL^{TM} reference library samples using 2X SYBR Green qPCR Mix (Thermo Fischer Scientific) according to the manufacturer's protocol. Universal forward and reverse primers (OpenDEL^{TM}) were used to target the 5' and 3' ends of the DEL sequence which are identical across all molecules. Thermocycling was conducted as follows on a QuantStudio5^{TM} (Applied Biosystems): 95 ˚C 10 min, ([95 ˚C 10s, 55 ˚C 10s, 72 ˚C 13s *collect signal] x 40 cycles). All solutions were prepared in DNase/RNase free UltraPure distilled water (Invitrogen) and each sample was performed in duplicate.

Next-generation sequencing

The selection output was amplified by PCR using Q5 Hot Start High-Fidelity 2X Master Mix (NEB, M0494L). Amplicons were purified by QIAGEN-MinElute PCR Purification Kit (QIAGEN, 28006) and quantified using Qubit DNA High-Sensitivity kit (Invitrogen, 32854) before library construction and sequencing. The library preparation was performed with Nextflex Rapid DNA-Seq Kit (BI00 Scientific, 5144-08) following the manufacturer's manual and libraries were sequenced on the Illumina NovaSeq platform (Illumina, USA) by HitGen. After sequencing, samples were decoded and analyzed as previously reported (10), and the results were visualized in DataWarrior (OpenMolecules) with each dimension representing one cycle of DEL construction.

Chemical feature enrichment analysis

The enrichment of a particular DEL chemotype (i.e., chemical feature) was assessed as previously described (10). Feature intensity enrichment scores were calculated as follows: sum of sequence counts for one feature divided by the average of the sum of sequence counts for all possible parallel features in the library. Chemical features that were highly enriched in the BI-bound β_1AR condition, potentially enriched in the apo-β_1AR sample, and minimally present in the G protein or β-arrestin transducer complex conditions were selected as potential hits. Signals that were present in the empty nanodisc control were excluded, as well as biotin-like binders. The structures of enriched features were further examined, and promiscuous features were cross-checked with the HitGen selection database (10).

Isothermal titration calorimetry (ITC)

Physical interaction of C11 with β_1AR bound to the high-affinity agonist BI-167107 was measured via ITC on a MicroCal PEAQ-ITC system (Malvern Panalytical) at 25 °C. Purified β1AR in maltose neopentyl glycol (MNG) (Anatrace; Maumee, OH) was dialyzed against 20 mM HEPES, pH 7.5, containing 100 mM NaCl, 0.01% MNG, and 0.001% cholesteryl hemisuccinate (HNMC). A volume of 200 µL of 15 µM β_1AR bound to BI-167107 (typical ligand concentration set at 300 µM in HNMC buffer) was loaded into the sample cell. To mitigate solubility limitations, the syringe solution containing 40 µL of C11 was gradually brought to a final concentration of 200 µM in HNMC buffer (with BI-167107 at 300 µM) through incremental dilution steps, followed by brief sonication (~2 min) and a high-speed spin (15 s at 12,000 × g). The clarified solution was then used for titrations, beginning with a 0.4 µL injection followed by nineteen 2.0 µL injections (at 180 s intervals) into the β_1AR–BI-167107 sample cell. During the experiment, the reference power was set to 7 $\mu cal \cdot s^{-1}$ and the sample cell was stirred continuously at 750 rpm. ITC raw data

were baseline-corrected, peak areas integrated and fitted using a one-site nonlinear least-squares model in MicroCal analysis software to obtain binding parameters including equilibrium dissociation constant (K_D), and stoichiometry (N).

FLIPR Ca^{2+} Assay (HEK293T)

HEK293T cells that endogenously express the M3 muscarinic acetylcholine receptor (M3R) (11) were plated at a density of 50,000 cells/well in poly-D-lysine-coated 96-well black, clear-bottom well plates and incubated overnight at 37 ˚C and 5% CO_2. Media was removed from plates and the cells were incubated with Ca^{2+}-sensitive fluorescent dye from the FLIPR Calcium 6 assay kit (Molecular Devices, San Jose, CA) for 2 hours according to the manufacturer's instructions. To study the effect of C11 on the Gq-coupled M3R, cells were pre-treated with vehicle (0.19% DMSO) or 30 μM C11 for 20 minutes. Fluorescence was measured with the FlexStation 3 microplate reader (Molecular Devices) for 2 minutes. Cells were stimulated with carbachol 20 seconds after starting fluorescence measurements. Baseline adjustments for each well were done by subtracting the average signal from the first 10 seconds of measurement. All Ca^{2+} responses were quantified as baseline-adjusted area under the curve of the fluorescent signal and presented as percent DMSO maximum.

Phospho-ERK assay

Evaluation of β_1AR-mediated activation of extracellular signal-regulated kinase (ERK) was performed as previously described with minor modifications (8). Briefly, 2.25 x 10^6 HEK293T cells maintained in growth media were seeded in a 10 cm dish, incubated overnight, and transfected with 2 ug human FLAG-β_1AR. After 24 hours, cells were re-plated in 6-well assay plates at a

density of 7.5 x 10^5 cells/well in growth media. Cells were starved for 3 hours in serum-free media (MEM supplemented with 0.1% BSA, 10 mM HEPES, and 1% P/S), pre-treated with vehicle (0.19% DMSO) or 30 μM C11 for 20 minutes at 37 ˚C along with 100 nM ICI-118,551 to block endogenous β_2ARs, and stimulated with serial concentrations of isoproterenol or carvedilol for 5 minutes. Cells were subsequently harvested in ice-cold lysis buffer (20 mM Tris pH 7.4, 137 mM NaCl, 20% glycerol, 1% Nonidet P-40, 2 mM sodium orthovanadate, 1 mM phenylmethylsulphonyl fluoride, 10 mM sodium fluoride, 10 μg/mL aprotinin, 5 μg/mL leupeptin, and phosphatase inhibitors) and rotated for 30 minutes at 4 ˚C. Cell lysates were separated on a 10% SDS-polyacrylamide gel and transferred to a polyvinylidene difluoride (PVDF) membrane. Blocked membranes were probed with anti-p44/42 MAPK (1:1000; Cell Signaling Technology, Danvers, MA) or anti-MAPK 1/2 (1:2000; Millipore, Burlington, MA) primary antibodies, and horseradish peroxidase (HRP)-conjugated secondary antibodies (1:3000; donkey anti-rabbit IgG, NA934V, Cytiva, Marlborough, MA). Following incubation with ECL chemiluminescent substrate (SuperSignal™, Thermo Fischer Scientific), immunoreactive bands were visualized with a ChemiDoc XRS+ imager (Bio-Rad, Hercules, CA). Densitometry of phospho-ERK was performed with ImageJ and normalized to total-ERK. The mean ± SEM of at least 3 technical replicates were plotted in GraphPad Prism and fit to a log(agonist) vs response (three parameter) model. Statistical analysis of the nonlinear curve fit (E_{max}) was evaluated by two-tailed t-test.

High-Performance Liquid Chromatography–Mass Spectrometry (HPLC-MS) Analysis

Stability analysis of C11 was performed on a 6224 TOF LC/MS system (Agilent Technologies), consisting of a 1200 HPLC (degasser, binary pump, thermostated column compartment, diode array detector (DAD)) coupled to a 6224 accurate-mass time-of-flight mass

spectrometer. The mass spectrometer was equipped with a Dual ESI source, and accurate mass data was obtained by internal calibration (reference ion 922.009798 m/z) using a secondary nebulizer to deliver the reference solution continuously. Positive-ion mass spectral data were acquired in full-scan mode over the range of 75-3200 m/z using the following source parameters: gas temperature 325 °C, gas flow 11 L/min, nebulizer pressure 33 psig, VCap 3500 V, and fragmented voltage 150 V. Aliquots of 10 mM C11 formulated in a mixture of DMSO/PEG400 (50%/50%, v/v) were incubated at 37 °C and withdrawn at different time points (0, 1, 6, 12, 24, 48, and 72 hours), flash-frozen in liquid nitrogen, and afterward transferred into a −80 °C freezer until HPLC-MS analysis. All C11 samples were first diluted with 50% acetonitrile in ddH2O to obtain 250 μM solutions. Subsequently, HPLC separations were achieved on an Agilent Zorbax SB-C18 column (2.1 × 150 mm I.D., particle size 3.5 μm) using a linear gradient of mobile phase B in A, a flow rate of 0.5 mL/min. Mobile phase A was prepared by combining 400 mL ultrapure water with 12 mL methanol and 1.2 mL formic acid. Mobile phase B was prepared by mixing 400 mL acetonitrile with 12 mL ultrapure water and 1.2 mL formic acid. The gradient program included an initial hold at 0% solvent B for 0.5 min, followed by a linear increase to 100% solvent B from 0.5-8 min, hold at 100% solvent B from 8.1-9 min, and re-equilibration back to 0% B for a total run time of 15 min. Samples were analyzed using a 1 μL injection volume. Target compound (C11) was confirmed by mass spectral data in positive ion mode, and U.V. spectra peaks (254 nm) were integrated to determine values for relative content and %-area purity (relative to t = 0 as 100% and then plotted as a function of time). Little to no impurities were detectable in C11. C11 had a purity of over 95% with a molecular ion peak, [M+H] at 561.34323 m/z.

Pharmacokinetics of C11 in mice

Male CD-1 mice (n=4; average body weight 28 g) were injected intraperitoneally with 10 mg/kg of C11 as 100 µL formulation containing 10% DMA, 40% PEG-300, 2% Tween 80, and 48% saline. For plasma, whole blood (~30 µL) was collected serially ("tail snip") at 5, 15, 30 minutes, 1, 3, 8, and 24 hours into vials containing 1 µL of 75 mg/mL K_2EDTA in water, and immediately frozen until the day of analysis. Heart tissue was harvested from 3 mice per time-point (30 minutes, 1 hour, and 3 hours) without saline perfusion. For liquid chromatography tandem-mass spectrometry (LC/MS/MS) analysis, 10 µL of each plasma sample was mixed with 20 µL of methanol/chloroform (1:1) fortified with 20 ng/mL C11-A (internal standard) and vigorously agitated in FastPrep FP120 apparatus (Thermo-Savant) at speed 4 for 45 seconds. After precipitation at -20 ºC for 15 min and centrifugation at 14,000 xg for 5 min at room temperature, 20 µL of supernatant was mixed with 20 µL of mobile phase A (see below), and 5 µL injected into LC/MS/MS system. For heart tissue analysis, the sample was homogenized with 3 parts water and 100 µL homogenate mixed with 10 µL of 20 ng/mL C11-A and 200 µL chloroform. After agitation and centrifugation, 150 µL of organic (lower) layer was evaporated to dryness (nitrogen stream), reconstituted with mobile phase A/mobile phase B (1:1) and 10 µL injected into LC/MS/MS system. LC/MS/MS (Agilent 1200 series HPLC and Sciex/Applied Biosystems API 5500 QTrap) was utilized to quantify C11. Analytical column: Agilent Eclipse Plus (C_{18}, 1.8 µm, 50 × 4.6 mm), at 40 ºC. Mobile phase: (A) 0.1% formic acid, 2% acetonitrile in water, (B) acetonitrile. Isocratic elution: 30% A, 70% B. Run time: 2 min. Mass spectrometer parameters (voltages, gas flow, and temperature) were optimized by infusion of 100 ng/mL of analytes in mobile phase at 10 µL/min using Analyst 1.6.2 software tuning module. The MS/MS (m/z) transitions used for quantification: 561.2/353.1 (C11), 575.4/353.1 (C11-A; internal standard). A set of calibrator samples in drug-

free matrix was prepared by adding appropriate amounts of pure analyte (C11) in 0.243 - 100 ng/mL range. The calibration samples were analyzed alongside the experimental samples. Accuracy acceptance criteria was 85% for each but the lowest level (80%, LLOQ = 0.243 ng/mL). Non-compartmental approach within WinNonlin (2.1) software was used for modeling of concentration/time data to calculate relevant pharmacokinetic parameters.

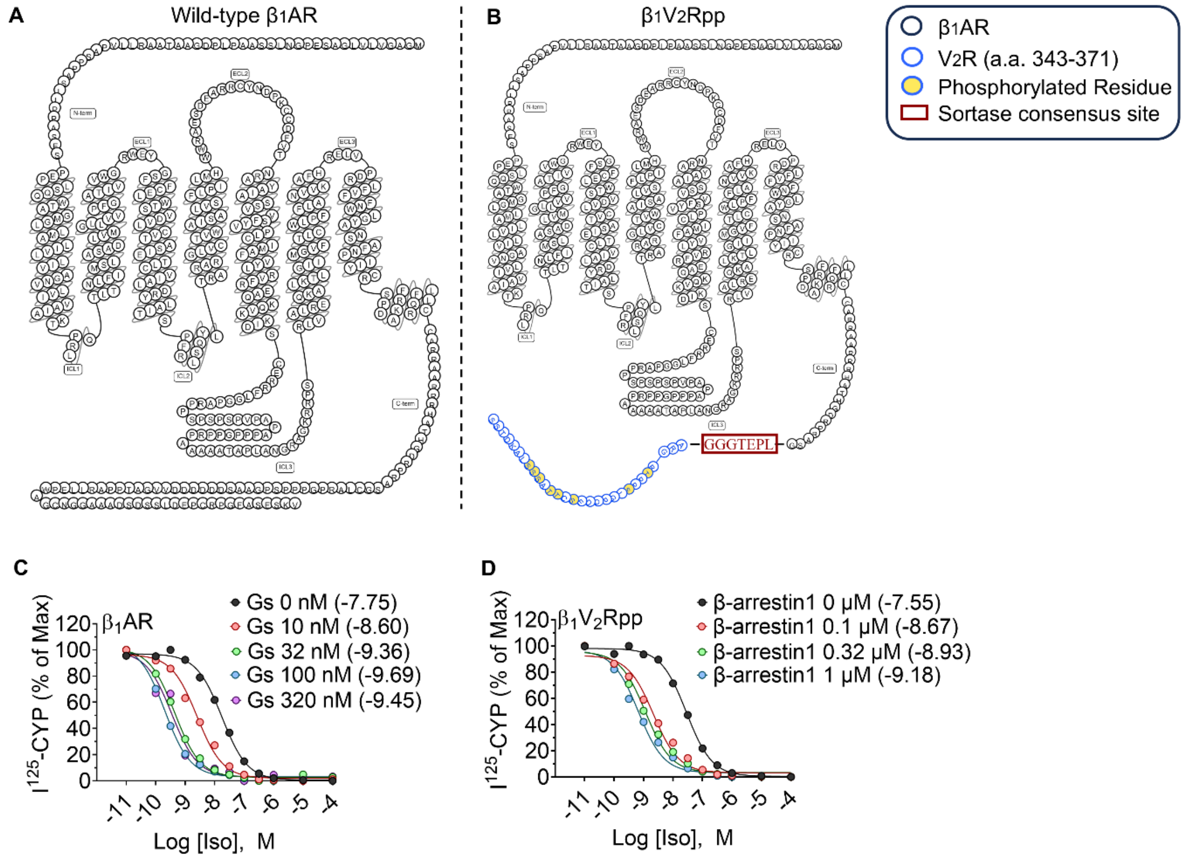

Supplemental Fig. 1. Generation and functional validation of β_1AR and β_1V_2Rpp nanodiscs. (A-B) Snake diagrams of wild-type (A) and chimeric (B) β_1ARs highlighting the cloning site of the sortase consensus sequence. The synthetic phospho-peptide corresponding to the V_2R was ligated to β_1AR truncated at G413 to generate β_1V_2Rpp. **(C-D)** Reconstituted nanodiscs containing β_1AR (C) or β_1V_2Rpp (D) were functionally validated using radioligand competition binding experiments in the presence of increasing concentrations of heterotrimeric G_s or β-arrestin1, respectively, to confirm transducer cooperativity. Values are presented as percent of maximum I^{125}-CYP binding. IC_{50} values (shown in parenthesis, in Molar units) were calculated from the nonlinear fit (one-site binding; GraphPad prism).

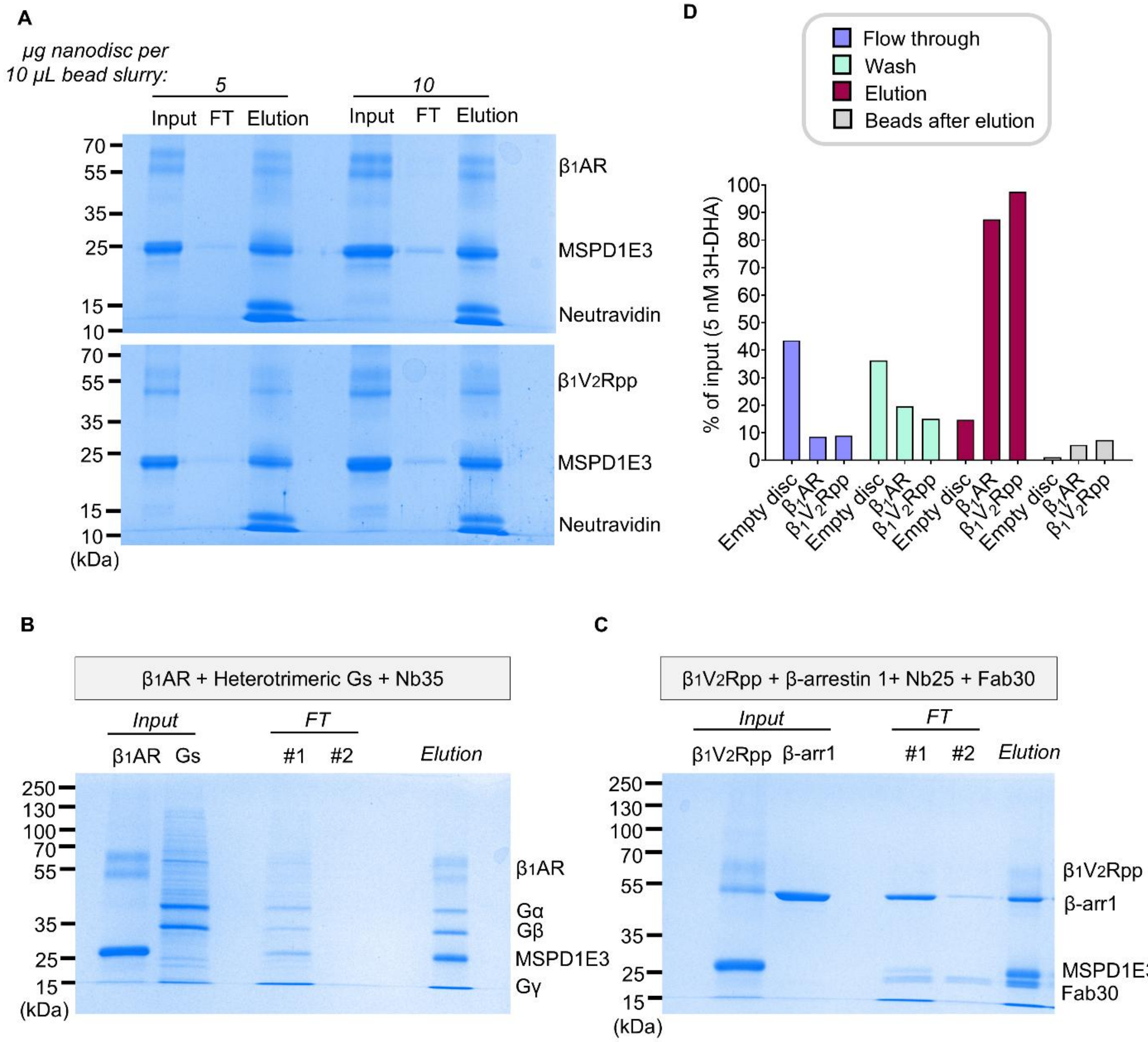

Supplemental Fig. 2. Optimization of DEL affinity selection protocol. (A) The immobilization efficiency of biotinylated β_1AR or β_1V_2Rpp nanodiscs was evaluated by incubating a fixed quantity of pre-washed high capacity neutravidin beads (10 uL) with increasing amounts of nanodisc (5-10 ug) at room temperature for 1 hour while rotating. After collecting flow through, the nanodisc-coated beads were washed three times and bound protein was eluted via boiling. Following SDS-PAGE and Coomassie Blue staining, a 1:1 ratio of receptor nanodisc (µg) to bead slurry (µL) was deemed optimal given minimal loss of nanodisc in the flow through. **(B-C)** To evaluate the integrity of the G protein and β-arrestin transducer complexes, β_1AR or β_1V_2Rpp nanodiscs were immobilized to neutravidin beads along with 20 µM of the high-affinity agonist, BI-167107 (BI), and a 1.2 molar excess of heterotrimeric G_s or β-arrestin1-mc, respectively. To enhance complex stability, β_1AR/G_s complexes were supplemented with Nb35 (2.5 molar excess with respect to β_1AR) and 0.05 U/mL apyrase, while the β_1V_2Rpp/β-arrestin1 complex was further stabilized with Nb25 and Fab30 (2.5 or 1.7 molar excess relative to β_1V_2Rpp, respectively). Following complex formation, the flow through was collected (FT #1) and the beads were washed three times with ice-cold binding buffer supplemented with 10 µM BI. To simulate incubation with DNA-encoded molecules, 1mg/mL salmon sperm DNA was applied to neutravidin-immobilized

β_1AR/Gs and β_1V_2Rpp/β-arrestin1 complexes in binding buffer containing 20 μM BI for 1 hour while agitating. The secondary flow through (FT #2) was collected, bound protein was eluted via boiling at 95 ˚C, and samples were loaded onto 10% SDS-polyacrylamide gels for analysis by Coomassie Blue to confirm the stability of receptor-transducer complexes throughout the selection protocol. **(D)** To validate the retention of small molecule ligands to the β_1AR or β_1V_2Rpp nanodiscs during screening, 5 nM of the radiolabeled orthosteric antagonist, ^{3}H-DHA, was incubated with neutravidin-immobilized nanodiscs and washed three times with ice-cold binding buffer. To elute, beads were resuspended in 1.5% Fos-choline in water and incubated at 37 ˚C (15 min) then 95 ˚C (15 min) while agitating. The elution procedure was repeated a second time, combined, and applied to 5 mL scintillation fluid for overnight incubation. ^{3}H-DHA counts are presented as percent of input. Empty nanodiscs were utilized as a control to assess non-specific binding.

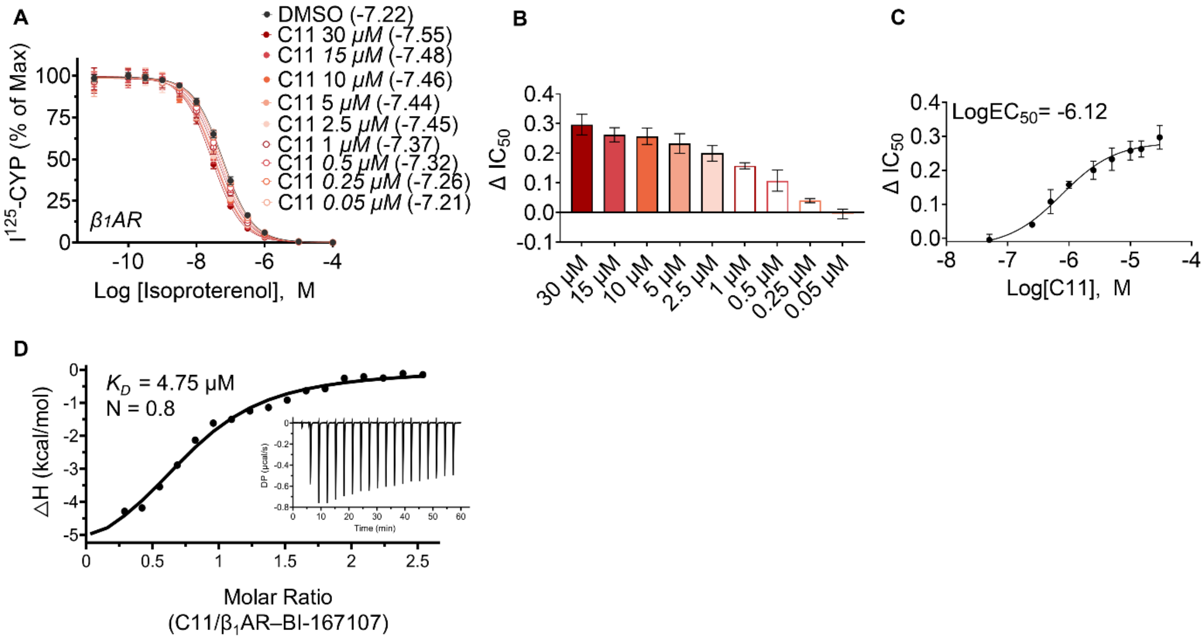

Supplemental Fig. 3. Evaluation of the affinity of C11 for the β_1AR. (A-C) Isoproterenol competition binding curves testing serial doses of C11 (A), and the corresponding IC_{50} shift quantifications (B) plotted as a function of [C11] (C) demonstrated that the binding affinity of C11 for the β_1AR is in the sub-micromolar range ($LogEC_{50}$ = -6.12 M); dose response curves are presented as percent of maximum I^{125}-CYP binding. IC_{50} values were calculated from the nonlinear fit (one-site binding; GraphPad Prism) and plotted as the difference between IC_{50} (DMSO) and IC_{50} (C11). Data points represent mean ± SEM of at least 3 independent experiments performed in duplicate. **(D)** Isothermal titration calorimetry (ITC) analysis of C11 binding to BI-bound β_1AR. Raw injection heats (insets) and fitted binding isotherms (solid lines) were modeled using a one-site independent binding model. Apparent equilibrium dissociation constant (K_D) and stoichiometry (N) values are shown as derived from the fit of an independent measurement.

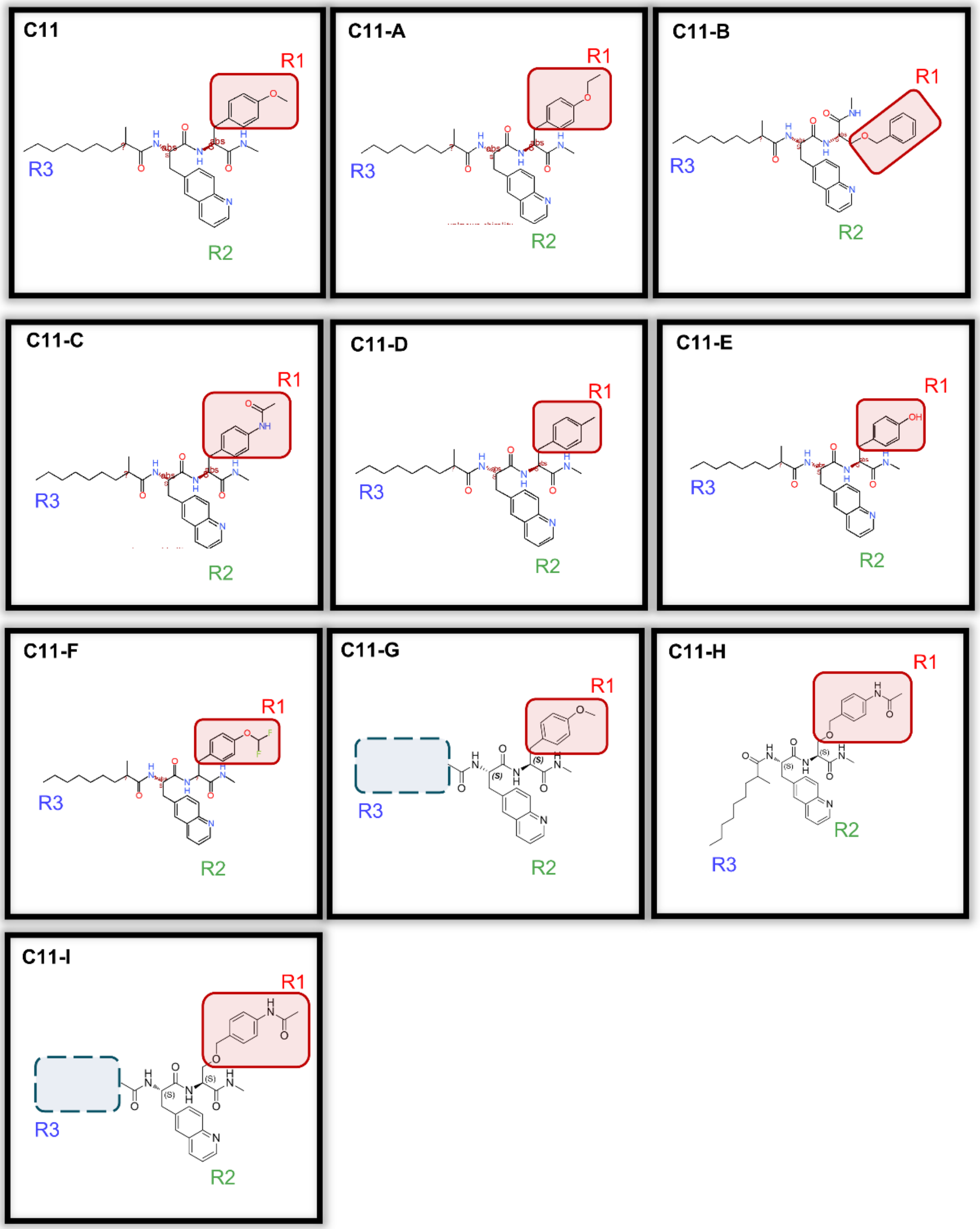

Supplemental Fig. 4. Chemical structures of C11 analogs A-I. The R1 chemical group (solid rectangle) and R3 chemical group (dashed rectangle) were modified in analogs C11-A through C11-I.

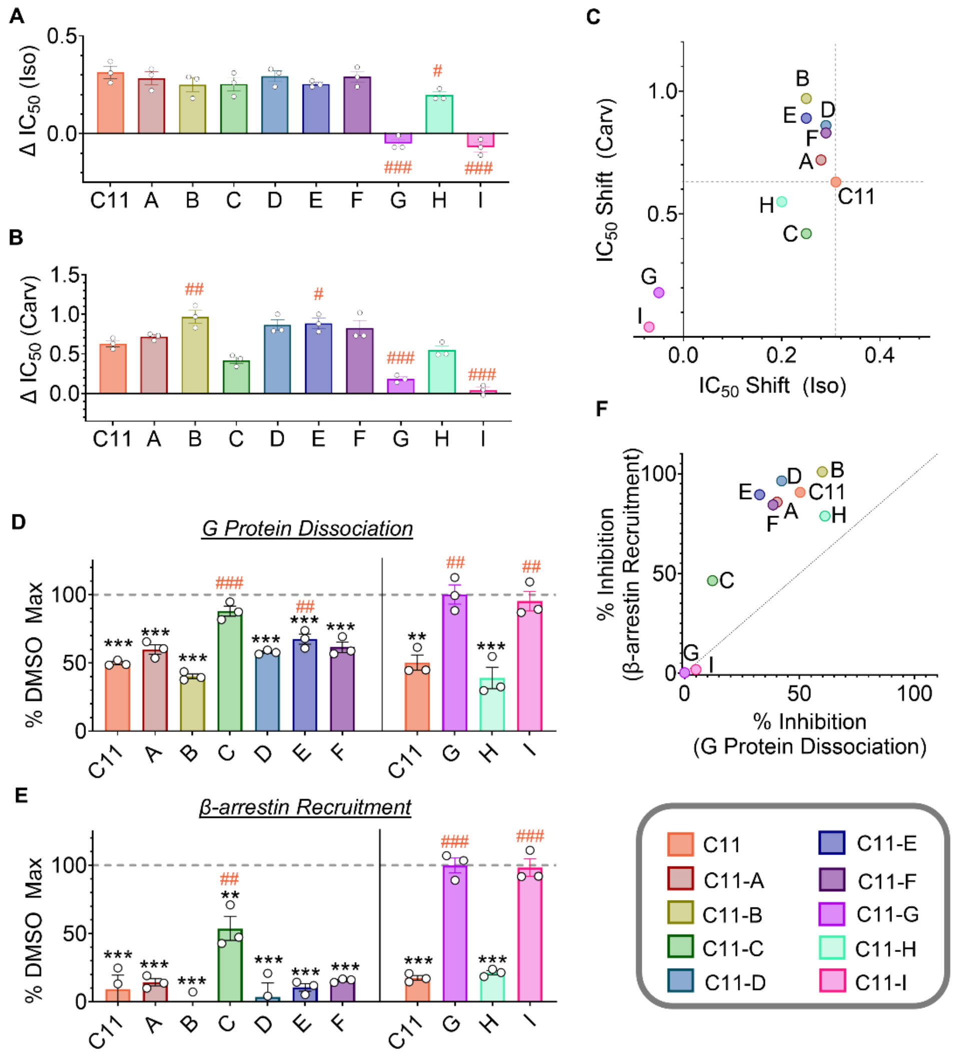

Supplemental Fig. 5. Effect of C11 analogs A-I on orthosteric ligand binding and β_1AR-mediated signaling. (A-C) β_1AR nanodiscs were incubated with a fixed amount of radiolabeled orthosteric antagonist, I^{125}-CYP, serial doses of unlabeled isoproterenol (A) or carvedilol (B), and either DMSO (0.19%) or 30 µM of C11 analogs. Quantification of IC_{50} log-shifts revealed that the ability of C11 to potentiate isoproterenol (Iso) or carvedilol (Carv) to the β_1AR is largely unaffected by modification of the R1 chemical group, whereas truncation of the R3 chemical group (C11-G and H) resulted in complete loss of cooperativity. IC_{50} values were calculated from the nonlinear fit (one-site binding; GraphPad prism) and plotted as the difference between IC_{50} (DMSO) and IC_{50} (C11). Data points represent mean ± SEM of at least 3 independent experiments performed in duplicate; one-way ANOVA, #p<0.05, ##p<0.01, ###p<0.001 (compared to C11). **(D-F)** Quantification of G protein dissociation (D) and β-arrestin recruitment (E) via BRET revealed that the ability of C11 to inhibit β_1AR-mediated signaling is largely unaffected by modification of the R1 group, whereas truncation of the R3 group (C11-G and H) resulted in complete loss of antagonistic function; data points represent mean ± SEM of at least 3 independent experiments performed in duplicate; curve fits were plotted using a log(agonist) three-parameter model in GraphPad Prism; net BRET ratios (emission of RLuc8/GFP) are baseline-subtracted

according to the non-linear fit of each treatment condition; one-way ANOVA, ##$p<0.01$, ###$p<0.001$ (compared to C11), **$p<0.01$, ***$p<0.001$ (compared to vehicle).

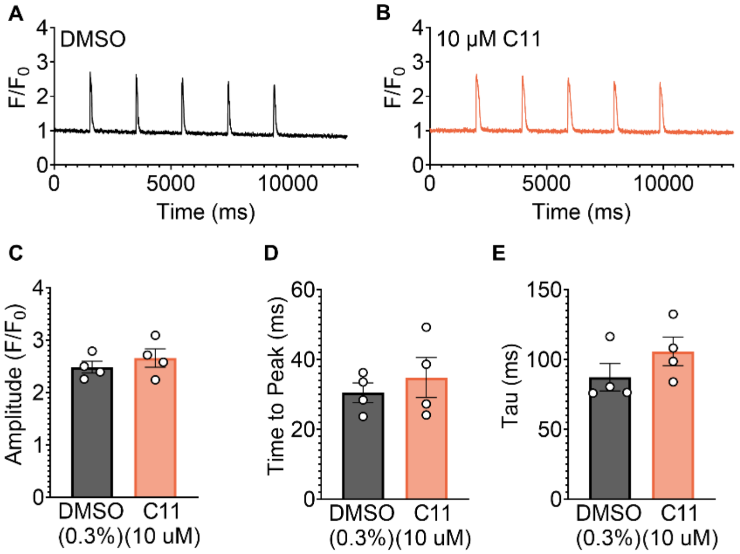

Supplemental Fig. 6. Effect of C11 on electrically stimulated Ca^{2+} transients in isolated $CSQ2^{-/-}$ cardiomyocytes. (A-B) Representative Ca^{2+} transients obtained from $CSQ2^{-/-}$ ventricular cardiomyocytes pre-treated with vehicle (A) or 10 µM C11 (B) during pacing at 0.5 Hz. **(C-E)** No significant differences in Ca^{2+} transient amplitude (F/F_0, C), time to peak (D), or the decay constant tau (E) were observed between groups; statistical significance was evaluated via t-test; data points represent biological replicates (n = 4 hearts, 11-13 cells per treatment per heart).

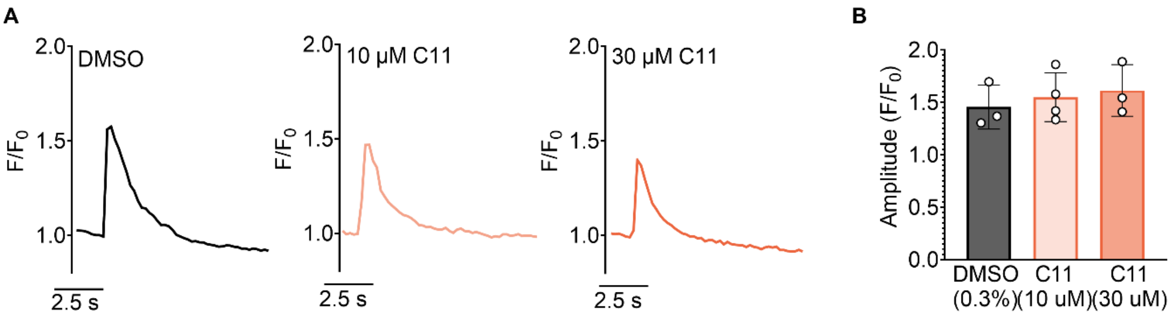

Supplemental Fig. 7. Effect of C11 on caffeine-stimulated Ca^{2+} release in isolated wild-type cardiomyocytes. (A-B) Caffeine-induced Ca^{2+} transients (A) were measured in quiescent wild-type cardiomyocytes pre-treated with vehicle or serial doses of C11 following treatment with 10 mM caffeine. No significant differences in Ca^{2+} transient amplitude (F/F_0, B) were observed between groups; statistical significance was evaluated via one-way ANOVA, data points represent biological replicates (n = 3 hearts, 2-6 cells per treatment per heart).

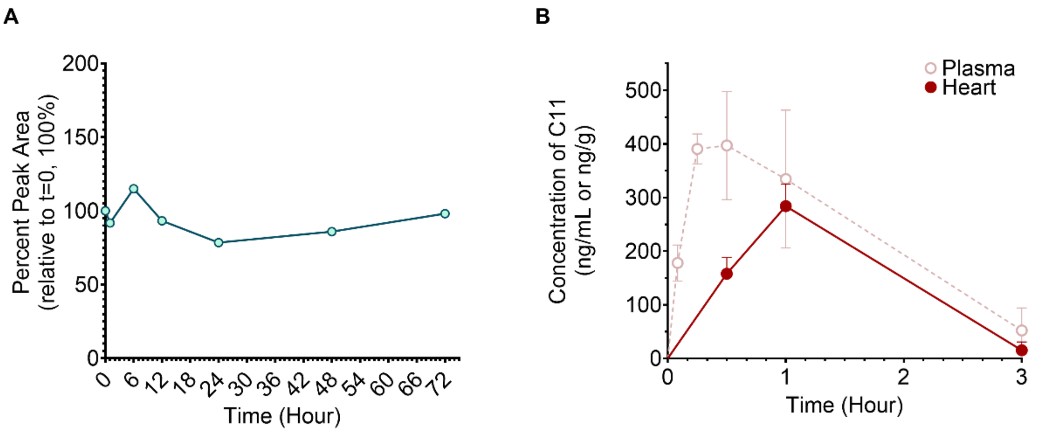

Supplemental Fig. 8. Stability of C11 in solution and *in vivo*. (A) C11 (10 mM) prepared in a vehicle solution (50% DMSO, 50% PEG-400) was incubated at 37 ºC for the indicated timepoints and applied to HPLC/MS to assess chemical stability. Quantification of peak area revealed that C11 is stable for at least three days at 37 ºC (n=1). **(B)** The pharmacokinetic profile of C11 prepared in a vehicle solution (10%DMA, 40% PEG-300, 2% Tween 80 and 48% saline) was obtained following intraperitoneal injection (10 mg/kg) in wild-type mice. C11 is detectable at the highest concentration between 30 minutes to 1-hour post-injection in plasma (ng/mL) and in heart tissue (ng/g); data points represent the mean ± SD of all biological replicates (n=3-4 mice).

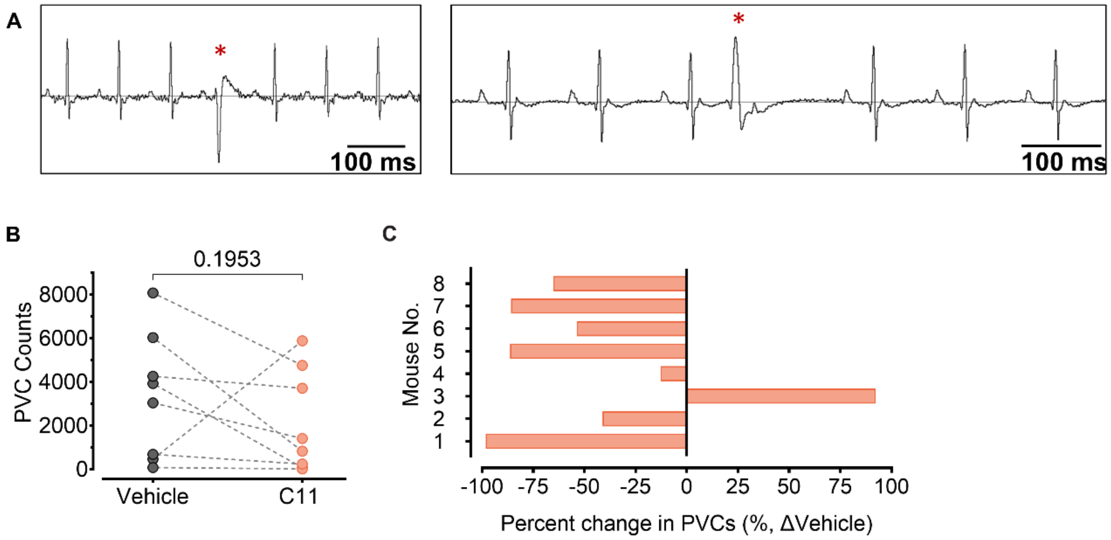

Supplemental Fig. 9. Effect of C11 on the incidence of PVCs during graded treadmill exercise. (A) Representative electrocardiograms obtained via continuous telemetric recording depict premature ventricular contractions (PVCs, red asterisks) in CSQ2$^{-/-}$ mice during physical exertion. **(B)** While most CSQ2$^{-/-}$ mice exhibited a reduction in the incidence of PVCs during exercise compared to when these same mice were pre-treated with vehicle, this was not statistically significant; data points represent biological replicates (n = 8 mice); Wilcoxon matched pairs signed rank test, p=0.1953. **(C)** The percent change in total PVCs after C11 treatment compared to vehicle was plotted for individual mice.

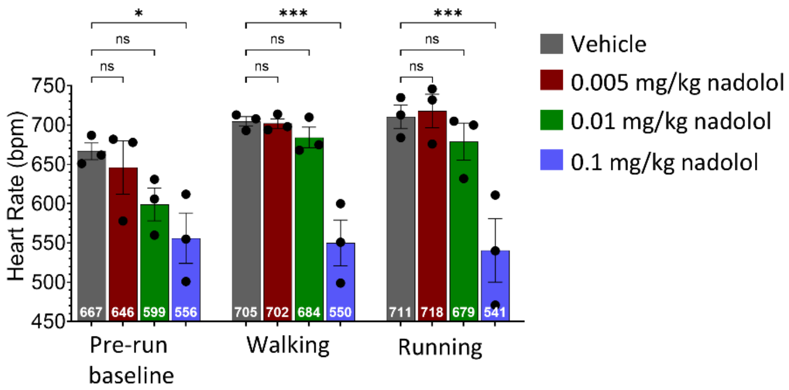

Supplemental Fig. 10. Effect of nadolol on heart rate during treadmill activity in wild-type mice. Heart rate was measured by wireless ECG telemetry in wild-type mice (n=3 per group) during three activity states: pre-run baseline, walking (2 cm/s), and running (10 cm/s). Each mouse received vehicle solution or nadolol at 0.005, 0.01, or 0.1 mg/kg in a repeated measures design, with a washout period of 7 days in between. Data are shown as mean ± SEM. Statistical analysis was performed using two-way repeated measures ANOVA followed by Dunnett's post hoc test comparing each dose to vehicle within each activity; *p< 0.05, ns, not significant.

Supplemental Table 1. IC_{50} values from radioligand competition binding assays.

Ligand	IC_{50} (DMSO), M	IC_{50} (C11), M	Δ IC_{50}
β_1AR Agonists			
Norepinephrine	-6.13±0.10	-6.57±0.10	0.44±0.02
Isoproterenol	-7.29±0.06	-7.63±0.07	0.33±0.00
Dobutamine	-5.65±0.05	-6.08±0.06	0.44±0.02
Epinephrine	-5.61±0.06	-5.89±0.06	0.28±0.01
β_1AR Antagonists			
Carvedilol	-8.74±0.07	-9.24±0.05	0.50±0.04
Bucindolol	-8.63±0.12	-8.98±0.07	0.35±0.06
Alprenolol	-7.88±0.08	-8.09±0.07	0.21±0.02
Atenolol	-5.62±0.08	-5.78±0.05	0.16±0.03
Metoprolol	-6.67±0.02	-6.69±0.03	0.02±0.01
Carazolol	-9.56±0.08	-9.56±0.06	0.01±0.02

β_1AR nanodiscs were incubated with a fixed amount of radiolabeled orthosteric antagonist, I^{125}-CYP, serial doses of unlabeled orthosteric ligand, and either DMSO (0.19%) or 30 μM C11. The resulting competition binding curves are plotted in Fig. 2A-B and corresponding IC_{50} shift quantifications in Fig. 2C. IC_{50} values (M, molar units) were calculated from the nonlinear fit (one-site binding; GraphPad prism) and are presented as the mean ± SEM of at least 3 independent experiments performed in duplicate. Statistical analysis of IC_{50} shifts is included in Fig. 2C.

Supplemental Table 2. Primer sequences utilized to clone BRET biosensors

Fragment	Forward or Reverse	Primer Sequence (5’-3’)
**β_1V_2R**		
β_1AR_G413	Forward	actcactatagggagacccaatgaagaccatcatcgccctgagctacatcttctgcct
β_1AR_G413	Reverse	tgcgtccccgggcgcccgaggcgcgcggccggtct
V_2R	Forward	gcgcctcgggcgcccggggacgcacccca
V_2R	Reverse	ctctagatgcatgctcgagctcacgatgaagtgtccttggccagggagg
**β_1V_2R-RLucII**		
β_1AR_G413	Forward	actcactatagggagacccaatgaagaccatcatcgccctgagctacatcttctgcct
β_1AR_G413	Reverse	tgcgtccccgggcgcccgaggcgcgcggccggtct
V_2R-RLucII	Forward	Gcgcctcgggcgcccggggacgcacccca
V_2R-RLucII	Reverse	Ctctagatgcatgctcgagcttactgctcgttcttcagcactctctccacgaagc

Fragments consisting of β_1AR truncated at residue G413 (β_1AR_G413) and V_2R were amplified and inserted into a linearized pcDNA3 empty vector to generate β_1V_2R via HiFi DNA assembly. Fragments consisting of β_1AR_G413 and V_2R-RLucII were amplified and inserted into a linearized pcDNA3 empty vector to generate β_1V_2R-RLucII via HiFi DNA assembly.

Supplemental Table 3. Effect of serial doses of C11 on contractility in isolated wild-type and β_1AR KO adult cardiomyocytes

Wild-type cardiomyocytes				
**Parameter**	**Treatment**			
	**DMSO (0.3%)**	**3 µM C11**	**30 µM C11**	**30 µM C11-G**
Baseline SL (µm)	1.72±0.01	1.76±0.02	1.81±0.02	1.70±0.01
Time to Peak (75%, ms)	62.28±1.73	62.02±1.46	62.74±3.83	55.80±2.44
Contraction Velocity (µm/sec)	-4.38±0.25	-3.84±0.13	-1.51±0.11	-5.02±0.10
Peak SL (µm)	1.46±0.01	1.54±0.02	1.71±0.02	1.43±0.02
Sarcomeric Shortening (%)	15.18±0.49	12.98±0.36	5.30±0.44	15.71±0.66
Time to Baseline (75%, ms)	112.33±5.65	108.94±8.43	142.84±12.53	113.36±9.53
Relaxation Velocity (µm/sec)	3.47±0.21	3.25±0.17	1.05±0.13	3.59±0.17

β_1AR KO cardiomyocytes				
**Parameter**	**Treatment**			
	**DMSO (0.3%)**	**3 µM C11**	**10 µM C11**	**30 µM C11**
Baseline SL (µm)	1.64±0.02	1.71±0.02	1.73±0.01	1.78±0.01
Time to Peak (75%, ms)	74.36±2.92	69.38±5.50	75.96±3.21	71.10±2.63
Contraction Velocity (µm/sec)	-3.01±0.36	-3.01±0.39	-2.20±0.20	-1.69±0.27
Peak SL (µm)	1.45±0.02	1.51±0.03	1.58±0.02	1.66±0.03
Sarcomeric Shortening (%)	12.03±0.83	11.35±0.78	8.88±0.61	6.48±1.13
Time to Baseline (75%, ms)	158.74±13.98	139.52±9.04	153.09±3.16	164.86±19.82
Relaxation Velocity (µm/sec)	2.07±0.28	2.24±0.26	1.63±0.12	1.21±0.36

Contractility parameters measured from isolated wild-type or $\beta_1AR^{-/-}$ cardiomyocytes during 1 Hz pacing (n= 4-7 hearts; 7-10 cells per treatment per heart). Statistical analysis of normalized sarcomeric shortening is included in Figure 6C-D. Values are represented ± SEM. SL, sarcomere length

References

1. Harding SD, Armstrong JF, Faccenda E, Southan C, Alexander SPH, Davenport AP, et al. The IUPHAR/BPS Guide to PHARMACOLOGY in 2024. *Nucleic Acids Res.* 2024;52(D1):D1438-D49.
2. Rasmussen SG, DeVree BT, Zou Y, Kruse AC, Chung KY, Kobilka TS, et al. Crystal structure of the beta2 adrenergic receptor-Gs protein complex. *Nature.* 2011;477(7366):549-55.
3. Staus DP, Hu H, Robertson MJ, Kleinhenz ALW, Wingler LM, Capel WD, et al. Structure of the M2 muscarinic receptor-beta-arrestin complex in a lipid nanodisc. *Nature.* 2020;579(7798):297-302.
4. Staus DP, Wingler LM, Choi M, Pani B, Manglik A, Kruse AC, et al. Sortase ligation enables homogeneous GPCR phosphorylation to reveal diversity in beta-arrestin coupling. *Proc Natl Acad Sci U S A.* 2018;115(15):3834-9.
5. Shukla AK, Manglik A, Kruse AC, Xiao K, Reis RI, Tseng WC, et al. Structure of active beta-arrestin-1 bound to a G-protein-coupled receptor phosphopeptide. *Nature.* 2013;497(7447):137-41.
6. Cahill TJ, 3rd, Thomsen AR, Tarrasch JT, Plouffe B, Nguyen AH, Yang F, et al. Distinct conformations of GPCR-beta-arrestin complexes mediate desensitization, signaling, and endocytosis. *Proc Natl Acad Sci U S A.* 2017;114(10):2562-7.
7. Tang Y, Hu LA, Miller WE, Ringstad N, Hall RA, Pitcher JA, et al. Identification of the endophilins (SH3p4/p8/p13) as novel binding partners for the beta1-adrenergic receptor. *Proc Natl Acad Sci U S A.* 1999;96(22):12559-64.
8. Wang J, Pani B, Gokhan I, Xiong X, Kahsai AW, Jiang H, et al. beta-Arrestin-Biased Allosteric Modulator Potentiates Carvedilol-Stimulated beta Adrenergic Receptor Cardioprotection. *Mol Pharmacol.* 2021;100(6):568-79.
9. Shenoy SK, McDonald PH, Kohout TA, and Lefkowitz RJ. Regulation of receptor fate by ubiquitination of activated beta 2-adrenergic receptor and beta-arrestin. *Science.* 2001;294(5545):1307-13.
10. Chen Q, Cheng X, Zhang L, Li X, Chen P, Liu J, et al. Exploring the Lower Limit of Individual DNA-Encoded Library Molecules in Selection. *SLAS Discov.* 2020;25(5):523-9.
11. Luo J, Busillo JM, and Benovic JL. M3 muscarinic acetylcholine receptor-mediated signaling is regulated by distinct mechanisms. *Mol Pharmacol.* 2008;74(2):338-47.
