# Supplementary material for: A positive allosteric modulator of the β1AR with antagonist activity for catecholaminergic polymorphic ventricular tachycardia
Source: J Clin Invest. 2025 Oct 16;135(24):e190252. doi: 10.1172/JCI190252 (PMC12700548; doi:10.1172/JCI190252)

Full unedited blots for Figure 4A

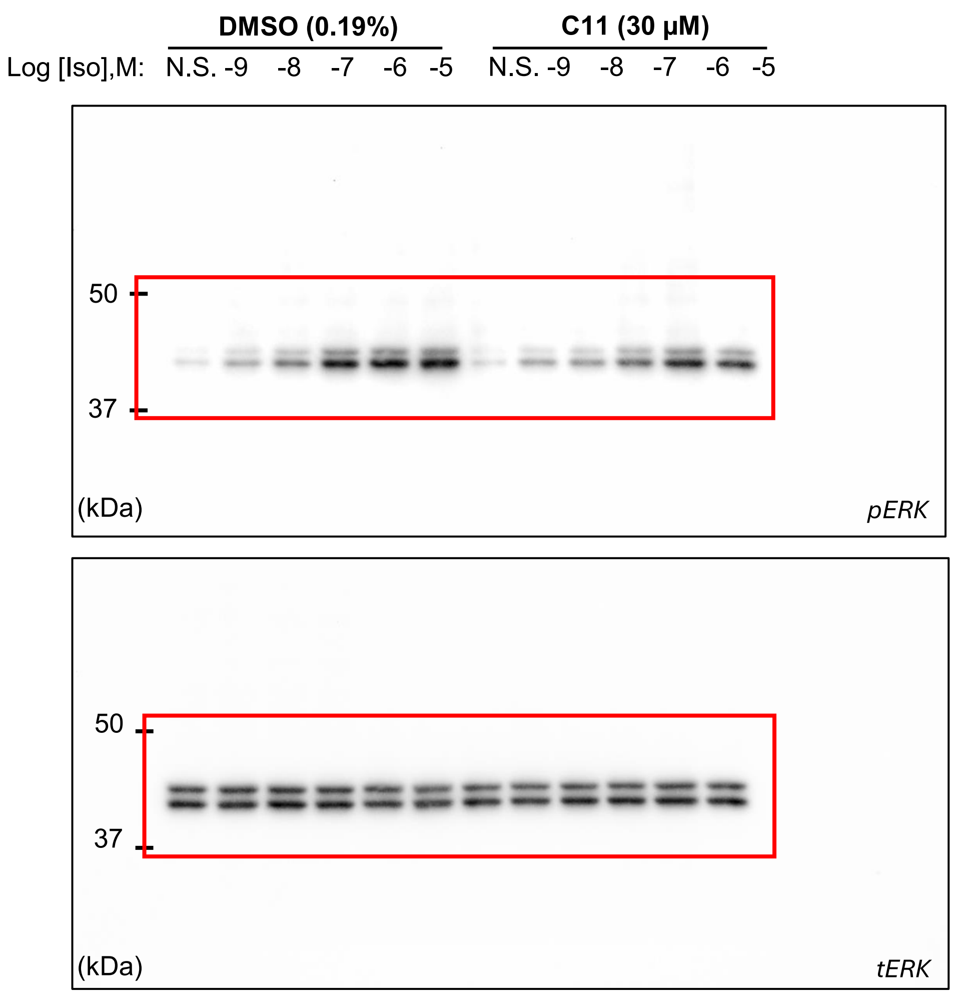

Full unedited blots for Figure 4B

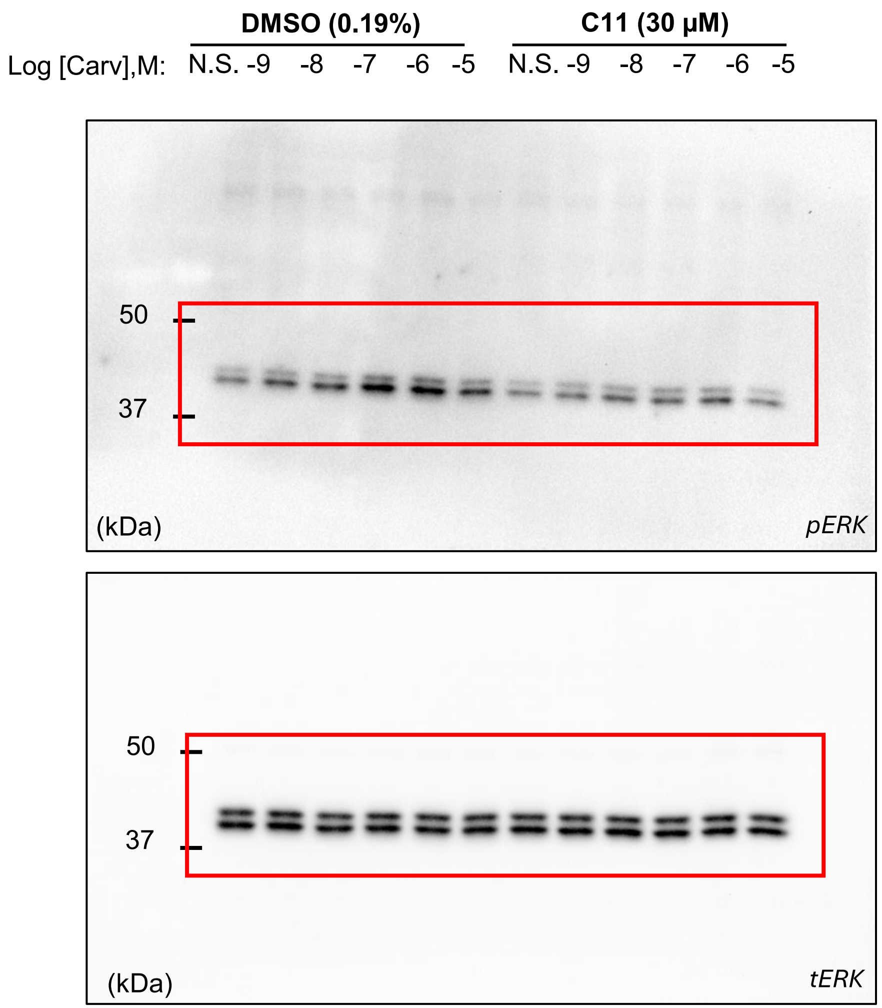

Representative lanes used in the figures are marked with red boxes

Full unedited blots for Figure 6H

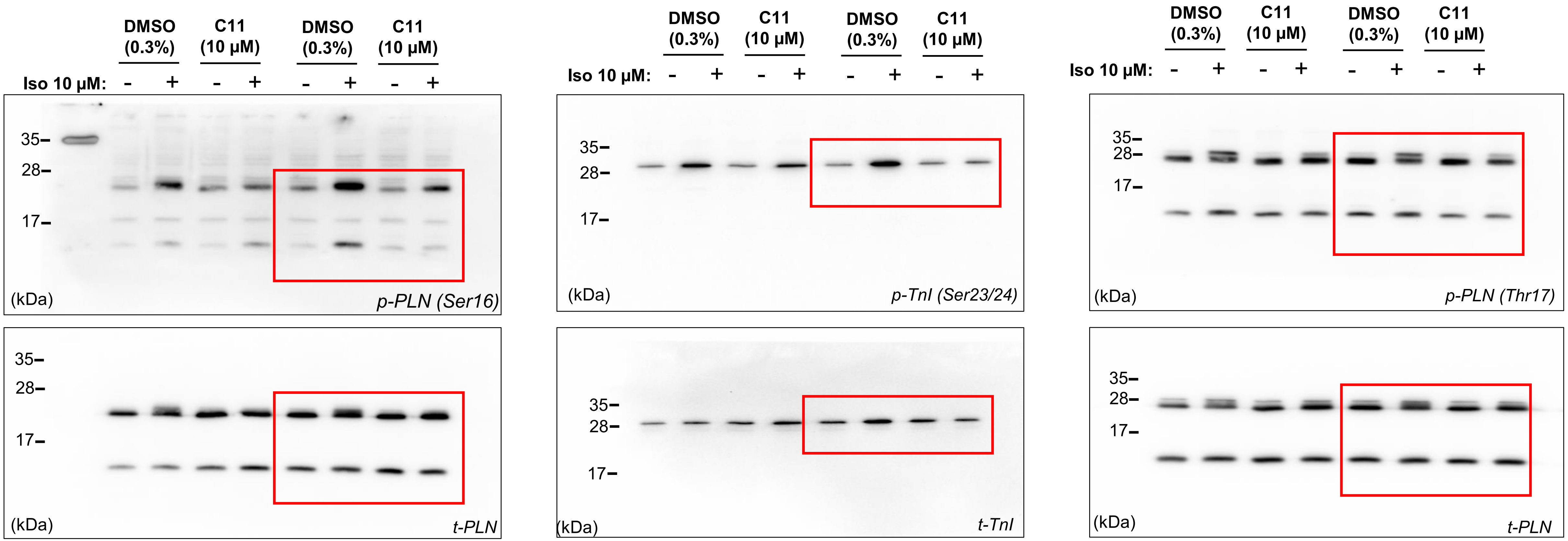

Representative lanes used in the figures are marked with red boxes

Full unedited gels for Supplemental Figure 2A

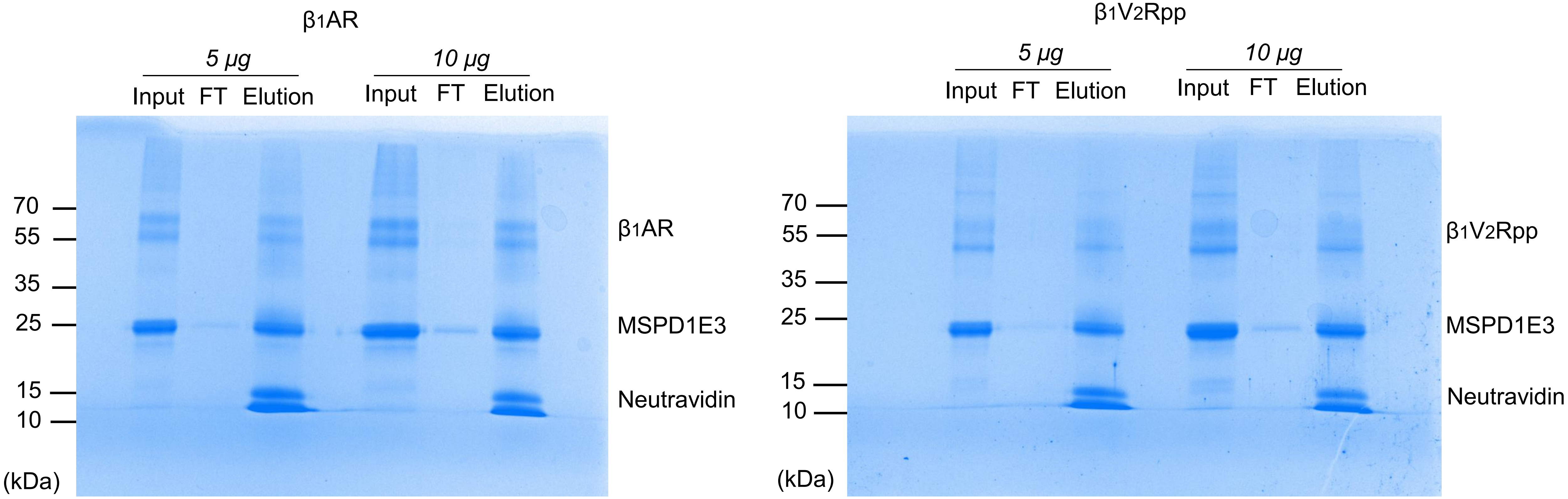

Full unedited gel for Supplemental Figure 2B

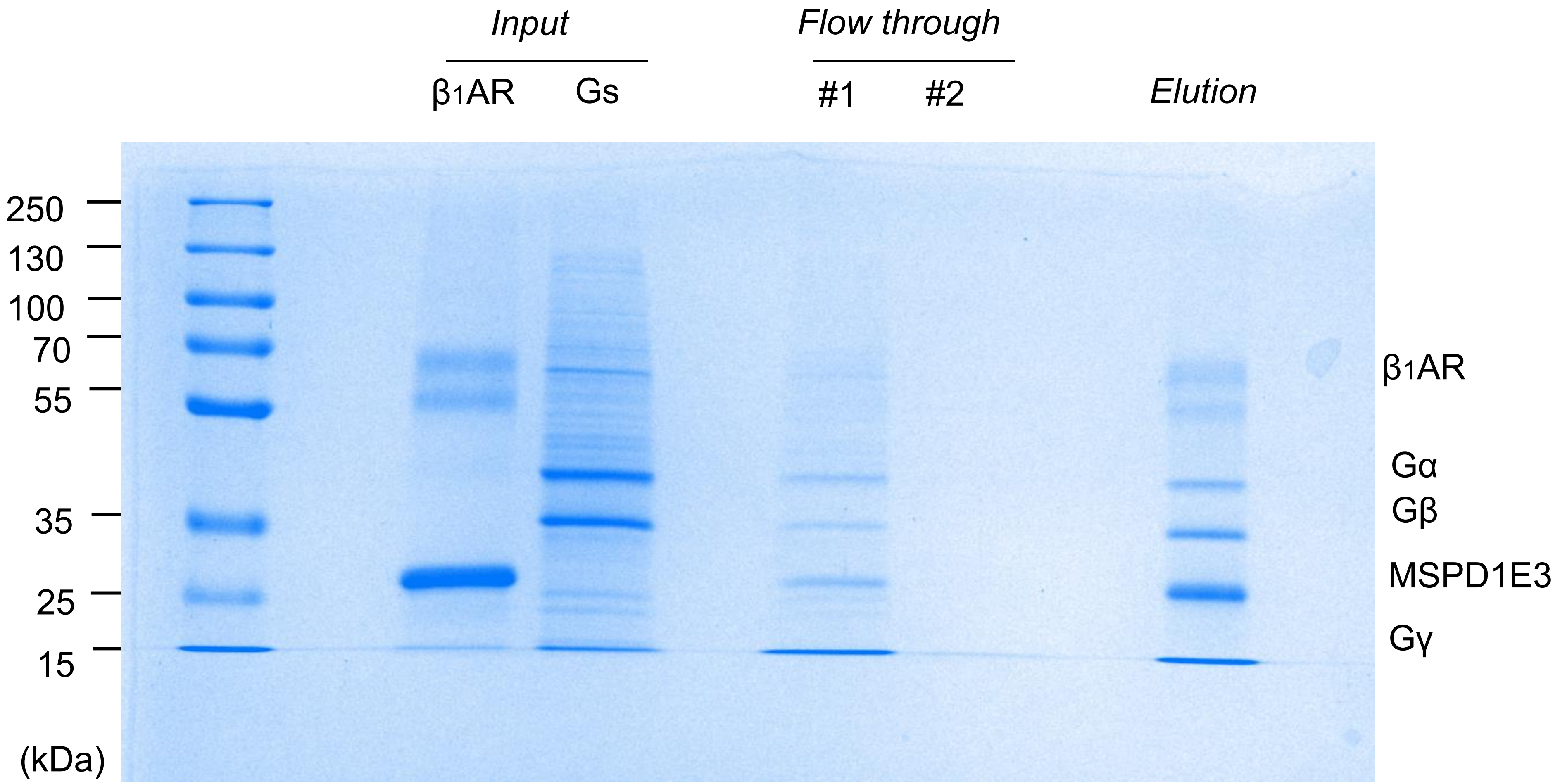

Full unedited gel for Supplemental Figure 2C

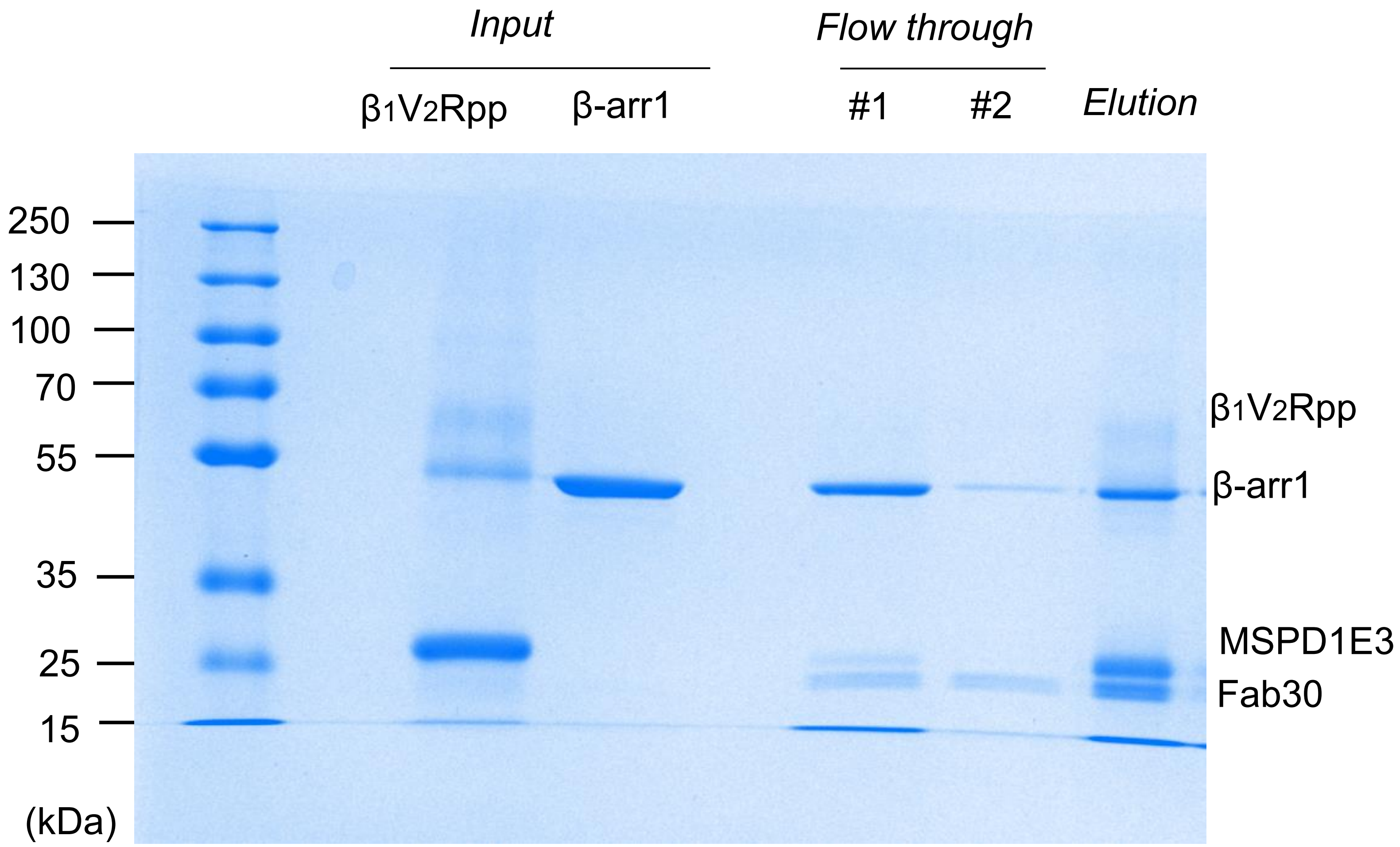

Supplement: Unedited blot and gel images [file jci-135-190252-s156.pdf]
